# BioID2-Based Tau Interactome Reveals Novel and Known Protein Interactions Associated with Multiple Cellular Pathways

**DOI:** 10.1021/acs.jproteome.5c00473

**Published:** 2025-09-05

**Authors:** Ahmed Atwa, Mohammed M. Alhadidy, Jared Lamp, Benjamin Combs, Nicholas M. Kanaan

**Affiliations:** † Department of Translational Neuroscience, College of Human Medicine, 12268 Michigan State University, Grand Rapids, Michigan 49503, United States; ‡ Neuroscience Program, 12268 Michigan State University, East Lansing, Michigan 48824 United States; § Integrated Mass Spectrometry Unit, College of Human Medicine, 12268 Michigan State University, Grand Rapids, Michigan 49503, United States

**Keywords:** BioID2, tau protein, mass spectrometry, immunoprecipitation, proximity ligation assay

## Abstract

Pathological inclusions composed of tau are hallmarks of neurodegenerative diseases termed tauopathies, the most common of which is Alzheimer’s disease. Accumulating evidence suggests that tau is involved in a multitude of physiological functions that are regulated, in part, by direct and/or transient protein interactions. Deciphering the tau interactome is critical for understanding the physiological and pathological roles of tau. This work aimed to identify potential tau interactors using the *in situ* protein labeling biotin identification (BioID2) method. Advantages of this approach include in-cell interactor labeling and an enhanced likelihood of detecting transient and/or weak interactions. We identified 324 potential tau interactors spanning multiple cellular compartments and pathways. We validated tau interactions with selected candidates using two independent approaches: proximity ligation assay and co-immunoprecipitation (co-IP) which included cytoskeletal proteins (MAP2 and MAP6), nucleus-associated proteins (FUS and prune1), and synaptic proteins (synapsin-1 and neurabin-2). Importantly, this approach revealed potential novel interactors that were not clearly identified by other interaction approaches such as co-IP. Thus, this approach is a powerful tool to identify potential members of the tau interactome via *in situ* labeling. This work helps expand our understanding of tau’s physiological roles, which may also advance our understanding of its role in neurodegenerative diseases.

## Graphical abstract

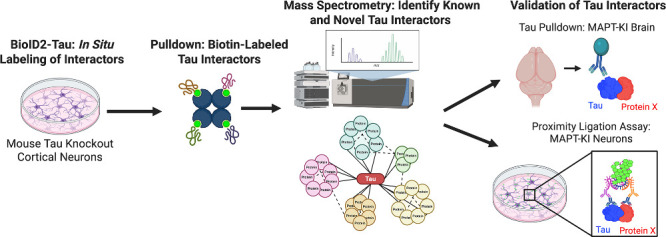

## Introduction

Tau was first defined as a microtubule-associated protein that is heat stable and regulates the assembly of microtubules.^1^ Physiological tau regulates several biological functions (e.g., regulating microtubule dynamics and axonal transport, among others) and is found in several neuronal subcellular compartments including the soma, dendrites, axons, synapses, and nucleus.^2–5^ Accumulation of abnormal forms of tau, such as aberrantly phosphorylated or misfolded tau, is a neuropathological hallmark and major contributor to neurodegeneration in Alzheimer’s disease (AD) and other tauopathies.^6^ Thus, tau is considered a key protein that regulates a multitude of physiological and pathophysiological functions in the brain.^2,7^

The cellular mechanisms that underlie microtubule-dependent and microtubule-independent functions of tau remain the focus of intensive research. A growing body of literature is focused on describing multifaceted direct and indirect protein–protein interactions as one way of studying the diverse functions of tau.^8,9^ Several studies characterized the tau interactome using antibody-based co-immunoprecipitation (co-IP) coupled with mass spectrometry in post-mortem human brain tissue, human cell lines, and animal models. Post-mortem human brain tissue studies identified tau protein interacting partners in the frontal cortex,^10,11^ hippocampus,^12^ and temporal gyri^13^ from AD brains. Other studies using co-IP approaches across several model systems identified interactome differences among the various tau isoforms.^14–16^ Alternative splicing of the human microtubule-associated protein tau (*MAPT*) gene yields six main isoforms of tau in the human brain comprising either zero, one, or two N-terminal inserts and three or four repeat domains in the microtubule binding region (0N3R, 1N3R, 2N3R, 0N4R, 1N4R, and 2N4R).^17^ Previous studies identified proteins in the tau interactome and discovered several isoform-specific differences in wild-type mouse brain tissue,^14^ human IMR-32 cells, or ReN VM cells expressing human tau isoforms (2N4R and 2N3R),^15^ and in human neuroblastoma SH-SY5Y cells expressing the 2N4R tau isoform^16^ using co-IP coupled with mass spectrometry. Similarly, other studies used these approaches to identify protein–protein interactions with a human tau construct consisting of aa 151–391 in transgenic SHR72 rats,^18^ P301L mutant human tau,^15,19^ endogenous mouse tau,^20^ or identified tau interacting proteins in tau seed competent fractions from PS19 transgenic mice.^21^

The use of antibody-based co-IPs to identify candidate interacting partners requires that the interaction be resistant to lysis and sample processing. Additionally, cell lysis can introduce nonphysiological conditions that may produce abnormal interactions. Therefore, this technique may be limited in its ability to effectively detect low-affinity and/or transient interactions.^22^ This approach is also somewhat limited by technical considerations like the antibody quality and the requirement that binding partners leave the IP antibody epitope available for binding.^22,23^ Over the past several years, enzyme-mediated proximity labeling emerged as a complementary approach that allows for *in situ* labeling and overcomes several limitations associated with the antibody-based approaches.^24–26^ Recent studies created fusion proteins between tau and ascorbate peroxidase (APEX2) or biotin identification (BioID2), both biotin ligase enzymes, allowing for *in situ* biotin labeling of proximal proteins in living cells.^27–29^ While APEX2 and BioID2 are both *in situ* proximity labeling approaches, an important distinction between them is that APEX2 provides a short-term snapshot of interactors (e.g., ∼1 min of labeling), whereas BioID2 enables longer-term interaction labeling (e.g., days) resulting in the accumulation of biotinylated interactors. Tracy et al. fused APEX2 to either the C-terminus or the N-terminus of the human tau 2N4R isoform and expressed the fusion constructs in human induced pluripotent stem cell-derived glutamatergic neurons (i^3^Neurons).^27^ Griffin and colleagues fused BioID2 to the C-terminus of wild type and mutant P301L 0N4R tau and expressed the constructs in human embryonic stem cell-derived NGN2 neurons.^29^ Prikas et al. fused BioID2 to the N-terminus of human tau 2N4R isoform and expressed the fusion protein in mouse wild type primary neurons and *in vivo* in wild type and tau knockout mice.^28^ In this study, BioID2 was individually fused to either the N-terminus or the C-terminus of full-length human 2N4R tau to compare the two interactomes in mouse tau knockout neurons. We performed mass spectrometry-based proteomics analysis to identify known and novel protein interactors and then validated several candidates using two independent approaches: co-IP and proximity ligation assay (PLA).

BioID2 is a mutated BirA biotin ligase that promiscuously biotinylates proteins in close proximity (approximately ≤ 10 nm).^30,31^ We created fusion proteins between BioID2 fused to either the N-terminus or the C-terminus of human tau 2N4R isoform (BioID2-Tau and Tau-BioID2 respectively) as well as two control constructs, the Myc-BioID2 (used as a control for BioID2-Tau) and the BioID2-HA (used as a control for Tau-BioID2). The BioID2 proteins were packaged in lentiviruses and expressed in tau knockout primary cortical neurons. We used two approaches to analyze the mass spectrometry data: qualitative protein identification of individual samples and label-free quantification (LFQ). We identified known and novel potential tau interacting proteins associated with multiple cellular compartments and complemented the BioID2 approach with two independent validation methods for a subset of the identified potential interactors (MAP2, MAP6, synapsin-1, neurabin-2, FUS, and prune1). Importantly, some of these interactions would not be identified using traditional co-IP approaches (e.g., MAP6 and neurabin-2). This work is distinct from prior *in situ* proximity labeling studies with tau in that we utilized BioID2 (on the N- or C-terminus of tau) to enable longer-term *in situ* labeling (compared to the APEX approach) in primary neurons without endogenous mouse tau expression (compared to the culture studies in Prikas et al. and Griffin et al.). Collectively, the BioID2 approach combined with independent validation approaches facilitated the detection of tau interactors and highlighted the diverse potential functions of tau associated with various cellular compartments (e.g., cytoskeleton, synapses, and nuclei).

## Experimental Section

### Animals

Human MAPT (tau) knock-in (MAPT KI) mice were obtained from Dr. Karen Duff at Columbia University with permission from the Saido group at Riken Center for Brain Science.^32–34^ Mouse tau knockout (TKO, strain no. 007251) mice were obtained from Jackson Laboratories. Homozygous breeding pairs were used to generate an in-house colony for both mouse lines, and timed-pregnant females were used to obtain fetuses for primary cortical neuron cultures. Animals were housed at Michigan State University Grand Rapids Research Center Vivarium in a 12 h light/dark cycle with access to food and water *ad libitum*. All animal work was conducted in compliance with federal, state, and institutional guidelines, and all procedures were approved by the Michigan State University Institutional Animal Care and Use Committee (protocol no. 202000068 and 202300048).

### BioID2 Constructs

The original BioID2 expression constructs, MCS-13X Linker-BioID2-HA (BioID2-HA, Addgene, no. 80899) and Myc-BioID2-13X Linker-MCS (Myc-BioID2, Addgene, no. 92308), were a kind gift from the Kyle Roux lab.^30^ The MCS-13X Linker-BioID2-HA plasmid was used to fuse BioID2 to the C-terminus of human Tau (hT40, 2N4R, 441 amino acids) to create a construct consisting of Tau-13xLinker-BioID2-HA (referred to as Tau-BioID2). The BioID2 control construct consisted of 13xLinker-BioID2-HA (referred to as BioID2-HA). The mycBioID2-13xLinker-MCS plasmid was used to fuse BioID2 to the N-terminus of tau to create the Myc-BioID2-13xLinker-Tau (referred to as BioID2-Tau) construct. The BioID2 control construct consisted of the Myc-BioID2-13xLinker (referred to as Myc-BioID2). BioID2 constructs feature a 13xLinker which is a serine-glycine repeat sequence that acts as a flexible spacer sequence to increase the biotinylation range of the BioID2 protein.^30^ The BioID2 constructs were then cloned into the pFIN vector (Addgene, no. 44352).^35^ All plasmid constructs were validated by restriction digestion and Sanger sequencing.

### Human Embryonic Kidney (HEK293T) Cell Culture

Complete Dulbecco’s Modified Eagle Medium (DMEM) was prepared by adding 500 mL of DMEM (Gibco, no. 11995073), 5% fetal bovine serum (FBS), and 1% penicillin-streptomycin (Sigma-Aldrich, no. P0781). Complete DMEM was filtered through a disposable vacuum 0.22 μm filter (Corning, no. 097611) and stored at 4 °C for up to one month. HEK293T cells were retrieved by thawing 2 × 10^6^ cells in 37 °C bead bath, centrifuged at 200 × *g* for 2 min, supernatant was discarded, and the cell pellet was resuspended in 5 mL complete DMEM. Cells were transferred to a T25 flask (ThermoScientific, no. 156340), and maintained for 4 days at 37 °C, 5% CO_2_ in a humidified incubator.

### Lentiviral Production in HEK293T Cells

Lentiviruses were created to express Tau-BioID2, BioID2-Tau, and the BioID2-HA and Myc-BioID2 controls. For each lentiviral preparation, 4 × 150 mm cell culture dishes (Corning, no. 430599) were plated at a density of 1 × 10^6^ cells/dish in 25 mL of complete DMEM. Culture dishes were incubated overnight in a humidified incubator. Two hours prior to transfection, DMEM was removed, and fresh DMEM was added. The following transfection mixture was then prepared for each 150 mm culture dish: 45 μg plasmid DNA (22.5 μg of the pFIN plasmid expressing the BioID2 protein of interest, 15 μg of pNHP lentiviral packaging vector (Addgene, no. 22500),^36^ and 7.5 μg of pHEF-VSVG lentiviral envelope vector (Addgene, no. 22501),^36^ 300 μL polyethylenimine transfection reagent and 6 mL of 150 mM NaCl. The transfection mixture was incubated for 20 min at room temperature, slowly added to each culture dish, and then transfected HEK293T cells were maintained overnight in a humidified incubator. Complete DMEM was substituted by freshly prepared viral medium (98% DMEM, 1% FBS, and 1% penicillin-streptomycin), and cells were maintained for 48 h. Medium containing lentiviruses was centrifuged at 675 × *g* for 5 min, and the supernatant containing lentiviruses was filtered through a 0.45 μm filter. Lentiviruses were harvested by layering on 20% sucrose solution, and ultracentrifugation at 82,700 × *g* for 2 h at 4 °C using Sorvall WX+ ultracentrifuge (ThermoScientific, no. 75000100). The lentiviral pellets were resuspended in 500 μL of sterile PBS, aliquoted, snap-frozen in crushed dry ice, and stored at −80 °C until use.

### Primary Cortical Neuron Cell Culture

Timed-pregnant female MAPT KI and TKO mice were euthanized by intraperitoneal injection of 100 mg/kg Fatal-Plus solution diluted in saline. Mouse pups were collected on embryonic day 18 (E18) and kept in ice-cold 0.9% saline. Fetal cortical tissues were isolated under a dissecting microscope, cut into small pieces, and collected in a tube containing ice-cold calcium- and magnesium-free solution (CMF; contains 1× DPBS, 1× amphotericin B (Gibco, no. 15290018), 1× gentamicin (Gibco, no. 15750060), and 10% glucose (Sigma-Aldrich, no. G8270).

Cortical tissue pieces were washed four times in CMF and incubated in 0.25% trypsin solution (Gibco, no. 15090046) for 15 min at 37 °C. Trypsin was removed, and cortices were washed two times in CMF. Trypsin inactivation solution (3 mL) composed of 2.1 mL Hank’s Balanced Salt Solution (Gibco, no. 24020117), 0.6 mL newborn calf serum (Gibco, no. 16010167), and 0.3 mL DNase solution (Worthington, no. LS002006) was added to the tissue. A homogeneous cell suspension was obtained by gentle trituration of the tissue through a series of progressively smaller needles (30 × 14-gauge needle, 30 × 15-gauge needle, 20 × 16-gauge needle, 20 × 18-gauge needle, and 15 × 21-gauge needle). Cell suspensions were gently layered onto 5 mL of sterile-filtered FBS and centrifuged at 200 × *g* for 5 min. Primary neuron cell pellets were gently resuspended in 1 mL of neurobasal media (NBM; Gibco, no. 21103049) supplemented with l-glutamine (2 mM, Gibco, no. 25030081), amphotericin B (2.5 μg/mL, Gibco, no. 15290026), B-27 Supplement (Gibco, no. 12587001), and gentamicin (50 μg/mL, Gibco, no. 15710064). Cell counts were determined using a Countess 3 automated cell counter (Invitrogen, no. AMQAX2000). Primary cortical neurons were plated at density of 600,000 cells per well in a poly-d-lysine-coated 6-well plate (Corning, no. 354413) and maintained in a humidified incubator at 37 °C and 5% CO_2_.

### Primary Neuron Lentiviral Transduction

TKO primary cortical neurons were treated with lentiviruses to induce the expression of BioID2-Tau, Tau-BioID2, or the respective Myc-BioID2 and BioID2-HA controls on 4 days *in vitro* (DIV4). Lentiviruses were diluted in complete NBM, and primary neurons were transduced at a multiplicity of infection of 200 (*n* = 3 biological replicates). The neuronal culture was maintained for 4 days to allow lentiviral transgene expression. Cultured neurons were supplemented with exogenous biotin (100 μM) and maintained for 4 days to facilitate the identification of transient/weak protein interactors over time. Lentivirus transduced neurons were maintained until collection for biochemical or immunofluorescence assays, as described below.

### SDS-PAGE and Western Blotting

On DIV12, cells were washed twice in 1× DPBS and protein lysates were collected in 1 mL of lysis buffer (50 mM Tris pH 7.4, 150 mM NaCl, 0.4% SDS, 1% NP-40, 1 mM EGTA, and 1.5 mM MgCl_2_) supplemented with protease inhibitors (pepstatin A (10 μg/mL, Sigma-Aldrich, no. P5318), leupeptin hydrochloride (10 μg/mL, Sigma-Aldrich, no. L9783), bestatin hydrochloride (10 μg/mL, Sigma-Aldrich, no. B8385), aprotinin (10 μg/mL, Sigma-Aldrich, no. A6279, and phenylmethanesulfonyl fluoride (1 mM, Sigma-Aldrich, no. 78830)), and phosphatase inhibitors (sodium pyrophosphate tetrabasic decahydrate (10 mM, Sigma-Aldrich, no. 71515), sodium orthovanadate (10 mM, Sigma-Aldrich, no. 450243), sodium beta-glycerophosphate pentahydrate (10 mM, Fisher, no. AAL0342522), and sodium fluoride (10 mM, Sigma-Aldrich, no. S7920). Lysates were sonicated 3 × 10 s and centrifuged at 12,000 × *g* for 10 min at 4 °C. Protein concentration was measured using Pierce rapid gold BCA protein assay kit (ThermoScientific, no. A53225) following the manufacturer’s instructions, and protein lysates were stored at −80 °C until use.

Lysates were prepared in 1× Laemmli sample buffer (2% Sodium dodecyl sulfate (SDS), 5% 2-mercaptoethanol, 10% glycerol, 0.002% bromophenol blue, 20 mM Tris-HCl pH 6.8) and heated to 95 °C for 5 min. Precast SDS-PAGE gels (Bio-Rad, no. 4561095) were used to separate proteins via electrophoresis in 1× running buffer (25 mM Tris base pH 8.3, 190 mM glycine, 0.1% SDS). Protein lysates (20 μg/lane) were run for 32 min at constant 250 V. Gels and nitrocellulose transfer membrane (Bio-Rad, no. 1620213) were soaked for 5 min in 1× transfer buffer (25 mM Tris pH 8.3, 192 mM glycine, and 20% methanol). Proteins were transferred from the gel onto a nitrocellulose membrane by running the transfer cassette for 50 min at a constant 400 mA. Membranes were blocked in 2% nonfat milk/Tris-buffered saline (TBS; 0.5 M Tris, 1.5 M NaCl, pH 7.4) for 1 h at room temperature. Membranes were incubated in primary antibodies diluted in blocking buffer overnight at 4 °C. The following primary antibodies were used: HA-tag antibody (1:1000, Cell Signaling Technology, no. 3724, RRID: AB_1549585), Myc-tag antibody (1:1000, Cell Signaling Technology, no. 2276, RRID: AB_331783). Membranes were washed three times in 1× TBS-T (50 mM Tris, 150 mM NaCl, pH 7.4, 0.1% Tween-20) and then incubated in secondary antibodies IRDye 680LT goat anti-mouse IgG (LI-COR Biosciences, no. 926-68020, RRID: AB_10706161) or IRDye 800CW goat anti-rabbit IgG (LI-COR Biosciences, no. 926–32211, RRID: AB_621843) diluted in blocking buffer (1:20,000). Membranes were washed three times in 1× TBS-T and imaged using a LI-COR Odyssey infrared system. Blot images were processed for publication using ImageStudio software (v5.2.5, LiCor Biosciences) and Adobe Illustrator 2023.

### Immunofluorescence

Primary neurons (treated as above) were fixed at DIV12 in 4% paraformaldehyde (PFA) for 20 min, washed three times in 1× TBS, and blocked for one h in blocking buffer (5% GS, 1% bovine serum albumin (BSA, Fisher Scientific, no. 9048-46-8), 0.2% Triton-X100 diluted in 1× TBS). Cells were incubated overnight in primary antibodies diluted in 2% GS, washed three times in 1× TBS, and incubated in secondary antibodies diluted at 1:500 in 2% GS for one h at room temperature. Cells were then washed three times in 1× TBS and counterstained using 4′,6-diamindino-2-phenylindole (DAPI; 0.5 μg/mL, ThermoScientific, no. D1306) diluted at 1:10,000 in 1× TBS. Primary antibodies used were HA-tag antibody (1:800, Cell Signaling Technology, no. 3724, RRID: AB_1549585), Myc-tag antibody (1:800, Cell Signaling Technology, no. 2276, RRID: AB_331783), and Tau13 antibody (1:10,000, RRID: AB_2721193, amino acids 8–9/13–21, Kanaan lab).^37,38^ Secondary antibodies used were Alexa Fluor 647 goat anti-mouse IgG1 (1:500, Thermo Fisher Scientific, no. A-21240, RRID: AB_2535809), Alexa Fluor 488 goat anti-rabbit IgG (1:500, Thermo Fisher Scientific, no. A-11008, RRID: AB_143165), Alexa Fluor 488 goat anti-mouse IgG2a (1:500, Thermo Fisher Scientific, no. A-21131, RRID: AB_2535771), and streptavidin Alexa Fluor 568 conjugate (1:500, Thermo Fisher Scientific, no. S-11226, RRID: AB_2315774). Fluorescent images were captured using the Nikon A1 + laser scanning confocal microscope equipped with a 40x oil-immersion objective and Nikon Elements AR software. Z-stack images (ten images/stack with 0.5 μm step size) were collected at 40× magnification using the same settings, and maximum intensity projection images were created. Images were processed for publication using Nikon Elements AR software and Adobe Illustrator 2023.

### Biotin–Streptavidin Affinity Pulldown

Ethanol-cleaned low retention microcentrifuge tubes (ThermoScientific, no. 3453) were placed in a magnetic separation stand. Streptavidin magnetic Dynabeads T1 (Invitrogen, no. 65602) were washed twice in 1 mL of lysis buffer, and then 1 mg of protein lysate containing biotinylated proteins (collected as described above) was added to the beads and rotated overnight at room temperature. The following day, unbound supernatant proteins were collected as the postpulldown sample and stored at −80 °C until further analysis. The beads containing bound biotinylated proteins were washed three times in lysis buffer and then resuspended in 1 mL of 50 mM Tris-HCl, pH 7.4. Then 900 μL of the resuspended beads was transferred to a new low retention tube for mass spectrometry analysis (described below). The remaining 100 μL was transferred to a new tube and was placed on a magnetic separation stand. The supernatant was discarded, and the beads were resuspended in an elution buffer containing 25 mM biotin prepared in lysis buffer.^39^ Efficient elution of biotinylated proteins was performed by competition with free biotin in the elution buffer and heating to 95 °C for 15 min. The tube was placed on a magnetic separation stand, the supernatant containing biotinylated proteins was transferred to a new tube, and Western blotting validation was performed as described above. Blots were incubated in IRDye 680LT Streptavidin (1:5000, LI-COR Biosciences, no. 926-68031) overnight at 4 °C. Blots were imaged using a LI-COR Odyssey infrared system and processed for publication using ImageStudio software (v5.2.5, LiCor Biosciences) and Adobe Illustrator 2023.

### Nanoscale Liquid Chromatography Coupled to Tandem Mass Spectrometry

The tube containing 900 μL of resuspended beads was placed on a magnetic separation stand, and the supernatant was discarded. The beads were washed six times in 25 mM ammonium bicarbonate (pH 8) and then resuspended in 150 μL of 25 mM ammonium bicarbonate/50% acetonitrile (ACN). On-bead protein digestion was performed by adding 100 ng of rLys-C (Promega, no. V1671) and incubating for 90 min at 37 °C, then adding 3 μg trypsin (Promega, no. V5280) and incubating for 16–18 h at 37 °C. The tubes were placed on a magnetic separation stand, and the supernatant was collected in a new low retention tube. Samples were dried to completion in a speed vacuum centrifuge at 30 °C before being resuspended in 50 μL of 25 mM ammonium bicarbonate/5% ACN.

Nanoscale liquid chromatography coupled MS/MS separations were performed with a Thermo Scientific Ultimate 3000 RSLCnano System. Peptides were desalted in-line using a 3 μm diameter bead, C18 Acclaim PepMap trap column (75 μm × 20 mm) with 2% ACN, 0.1% formic acid (FA) for 5 min with a flow rate of 5 μL/min at 40 °C. The trap column was then brought in line with a 2 μm diameter bead, C18 EASY-Spray column (75 μm × 250 mm) for analytical separation over 60 min with a flow rate of 350 nL/min at 40 °C. The mobile phase consisted of 0.1% FA (buffer A) and 0.1% FA in ACN (buffer B). The separation gradient was as follows: 5 min desalting, 40 min 4–40% B, 2 min 40–65% B, 2 min 65–95% B, 7 min 95% B, 1 min 95–4% B, 3 min 4% B. One microliter of each sample was injected. Top 20 data-dependent mass spectrometric analysis was performed with a Q Exactive HF-X Hybrid Quadrupole-Orbitrap Mass Spectrometer. MS1 resolution was 60K at 200 *m*/*z* with a maximum injection time of 45 ms, AGC target of 3e6, and scan range of 300–1500 *m*/*z*. MS2 resolution was 30K at 200 *m*/*z*, with a maximum injection time of 54 ms, AGC target of 1e5, and isolation range of 1.3 *m*/*z*. HCD normalized collision energy was 28. Only ions with charge states from +2 to +6 were selected for fragmentation, and the dynamic exclusion was set to 30 s. The electrospray voltage was 1.9 kV at a 2.0 mm tip to inlet distance. The ion capillary temperature was 280 °C, and the RF level was 55.0. All other parameters were set as a default.

### Mass Spectrometry Protein Identification

RAW mass spectrometry files were identified using Thermo Scientific Proteome Discoverer software (version 2.5) and Sequest against the reviewed *Mus musculus* Uniprot proteome database (UP000000589, 25,285 unique sequences) additionally including trypsin (P00761), Lys-c (Q7M135, Q02SZ7), human tau (P10636-(0-9)), biotin ligase (3EFR_1), and streptavidin (P22629, Q53532, Q53533). Enzyme specificity was set to trypsin, allowing up to 2 missed cleavages with an MS1 tolerance of 10 ppm and a fragment tolerance of 0.02 Da. Oxidation (M), biotinylation (K), acetylation (protein N-term), methionine loss (protein N-term), and biotinylation (protein N-term) were set as dynamic modifications. Peptide and protein false discovery rates (FDR) were 1% with the threshold determined via decoy search using the Percolator algorithm. At least two peptide identifications were required per protein identification. Peptide confidence was set to “High”. All other parameters were set as default. Mass spectrometry RAW files from each experimental sample (*n* = 3) or control sample (*n* = 3) were analyzed by qualitative protein identification or LFQ (see details below). For the qualitative method, proteins identified in only one independent experimental replicate were excluded from further analysis, as were all keratins, digestion enzymes, and proteins directly associated with the pulldown. To identify potential tau protein interacting partners, protein lists from Tau-BioID2 and BioID2-Tau experiments were compared to the respective controls, BioID2-HA and Myc-BioID2. Proteins common to the controls were removed, and the curated Tau-BioID2 and BioID2-Tau lists were merged to generate the comprehensive list of potential tau interactors. An additional, more stringent analysis was conducted by including only proteins identified in all three replicates of the Tau-BioID2 or BioID2-Tau experimental conditions, which were then compared to proteins in all three replicates of the respective BioID2-HA or Myc-BioID2 controls. Again, proteins in controls were removed, and the curated Tau-BioID2 and BioID2-Tau lists were merged to compile the list of potential tau interactors.

### Label-Free Quantification of Identified Proteins

LFQ was performed using the Precursor Ions Quantifier node. Unique and Razor peptides were considered for quantitation. Precursor abundance was based on the peak intensity. Protein abundance was normalized by the total peptide amount. The protein abundance ratio was calculated using pairwise ratios (excluding modified peptides), whereby the median peptide ratio was selected as the protein abundance ratio. The following criteria were used to define proteins as Tau-BioID2 or BioID2-Tau interactors: 1) being identified in at least two of the independent experimental replicates, and 2) being detected at ≥3-fold increase compared to the respective BioID2 control. Proteins identified in only one independent replicate as well as contaminant keratins, digestion enzymes, and proteins directly associated with the pull-down were removed from further analysis.

### Functional Protein Association Networks

Functional protein–protein interaction networks were visualized by the Search Tool for the Retrieval of Interacting Genes and Proteins (STRING) analysis. STRING V12.0 was used to visualize the protein interactome network including both functional associations retrieved from published databases, text mining, computational prediction methods, and physical interactions retrieved from experimental data (genetic, biochemical, and biophysical techniques), with a minimum required confidence interaction score set at 0.7 and above (high confidence interaction).^40^ STRING V12.0 was used to perform Markov Clustering (MCL)^41^ with an inflation value of 3.0 to reduce the cluster size. STRING V12.0 was used to perform the Gene Ontology (GO) enrichment analysis and KEGG pathways analysis. GO cellular component, molecular function, biological process, and KEGG pathways were performed, showing annotations with FDR ≤ 0.05.

### Co-Immunoprecipitation of Candidate Tau Interacting Partners

The MS-identified interacting partners were validated in MAPT KI mouse brain tissue using co-IP. Cortical tissue lysates were collected from three month old saline perfused MAPT KI mice. Mice were injected intraperitoneally with sodium pentobarbital (50–100 mg/kg) and were then transcardially perfused with 0.9% saline with heparin (10,000 units/L). Cortices were collected in ice-cold lysis buffer containing 25 mM HEPES pH 7.4, 50 mM potassium acetate, 2 mM magnesium acetate, 1 mM EGTA, 1 mM DTT, and 10% glycerol and supplemented with protease inhibitors (pepstatin A (10 μg/mL, Sigma-Aldrich, no. P5318), leupeptin hydrochloride (10 μg/mL, Sigma-Aldrich, no. L9783), bestatin hydrochloride (10 μg/mL, Sigma-Aldrich, no. B8385), aprotinin (10 μg/mL, Sigma-Aldrich, no. A6279, and Phenylmethanesulfonyl fluoride (1 mM, Sigma-Aldrich, no. 78830) and phosphatase inhibitors (sodium pyrophosphate tetrabasic decahydrate (10 mM, Sigma-Aldrich, no. 71515), sodium orthovanadate (10 mM, Sigma-Aldrich, no. 450243), sodium beta-glycerophosphate pentahydrate (10 mM, Fisher Scientific, no. AAL0342522), and sodium fluoride (10 mM, Sigma-Aldrich, no. S7920). Cortices were homogenized using a Dounce homogenizer and centrifuged at 12,000 × *g* for 10 min at 4 °C. Supernatant containing protein lysate was collected, and protein concentration was measured using Pierce rapid gold BCA protein assay kit (ThermoScientifc, no. A53225) according to manufacturer’s instructions. Streptavidin magnetic Dynabeads T1 (Invitrogen, no. 65602) were washed three times in 1× PBS and incubated for 1 h at room temperature with either biotinylated Tau13 antibody (30 μg) or biotinylated IgG isotype control (30 μg, Rockland, no. 010-0602, RRID: AB_840751). Coated magnetic Dynabeads were then washed four times in wash buffer (lysis buffer, 0.2% Triton-X100 (Bio-Rad, no. 161-0407), and 0.1% BSA). Protein lysates (1 mg) were then incubated with magnetic Dynabeads coated with either biotinylated Tau13 or biotinylated IgG isotype control for two h at room temperature. Supernatant was collected as the postimmunoprecipitation sample, and beads were washed four times in wash buffer (lysis buffer and 0.2% Triton-X100). Coated magnetic Dynabeads were then incubated in elution buffer (100 mM glycine-HCL, and 2 M urea pH 2.5) for 20 min to elute the antibody-bound proteins. Potential tau interacting proteins were validated by probing for them in the Tau13 elution samples using SDS-PAGE and Western blotting (as above). Primary antibodies used in co-IP experiments for validation included MAP2 (1:1,000, Cell Signaling Technology, no. 8707, RRID: AB_2722660), MAP6 (1:500, Cell Signaling Technology, no. 4265, RRID: AB_2140993), synapsin-1 (1:1,000, Cell Signaling Technology, no. 5297, RRID: AB_2616578), neurabin-2 (1:500, Bioss, no. BS-12146R), prune1 (1:500, Proteintech, no. 18537-1-AP, RRID: AB_2172338), and FUS (1:1000, Cell Signaling Technology, no. 67840). Secondary antibodies used were IRDye 680LT goat anti-mouse IgG1 (LI-COR Biosciences, #926-68050, RRID: AB_2783642) or IRDye 680LT goat anti-rabbit IgG (LI-COR Biosciences, no. 926-68021, RRID: AB_10706309) diluted in the blocking buffer (1:20,000). Blots were imaged using LI-COR Odyssey infrared system and processed for publication using ImageStudio software (v5.2.5, LiCor Biosciences) and Adobe Illustrator 2023.

### Proximity Ligation Assay

Potential interacting partners were also validated using PLA in cultured primary cortical neurons using the Duolink In Situ Fluorescence Protocol (Sigma) with minor modifications. E18 primary cortical neurons from MAPT KI or TKO mice were cultured at a density of 40,000 cells per well in 18 well chamber slides (Ibidi, no. 81817) precoated with poly-d-lysine (Sigma-Aldrich, no. P7886). On DIV12, primary neurons were fixed in 4% PFA in 1× cytoskeleton buffer (10 mM MES, 138 mM KCl, 3 mM MgCl2, and 4 mM EGTA, pH 6.1) for 20 min and blocked in 2% GS (VWR, no. 10152–212), 1% BSA, 0.2% Triton-X100 (Bio-Rad, no. 161-0407) prepared in 1× TBS for 1 h at room temperature. Cultured neurons were then incubated in primary antibodies diluted in 2% GS overnight at 4 °C. Tau13 primary antibody (1:100,000) was used in combination with MAP2 (1:30,000), synapsin-1 (1:10,000), neurabin-2 (1:300), prune1 (1:800), or FUS (1:2000). R1 primary antibody (1:30,000, RRID: AB_2832929, rabbit polyclonal pan-tau antibody, Kanaan lab) was used in combination with MAP6 (1:100, Cell Signaling Technology, no. 4265, RRID: AB_2140993). After primary antibody incubation, cultured cells were washed four times for 5 min in Wash Buffer A (Sigma-Aldrich, no. DUO82049). Combinations of either Duolink In Situ PLA Probe Anti-Mouse PLUS (Sigma-Aldrich, no. DUO92001, RRID: AB_2810939) and Duolink In Situ PLA Probe Anti-Rabbit MINUS (Sigma-Aldrich, no. DUO92005, RRID: AB_2810942) PLA probes or Duolink In Situ PLA Probe Anti-Rabbit PLUS (Sigma-Aldrich, no. DUO92002, RRID: AB_2810940) and Duolink In Situ PLA Probe Anti-Mouse MINUS (Sigma-Aldrich, no. DUO92004, RRID: AB_2713942) were applied. PLA probes diluted at 1:20 in 2% GS were applied to the cells for 1 h at 37 °C in a humidified chamber. Cells were washed four times for 5 min in Wash Buffer A (Sigma, no. DUO82049), and ligation solution was applied for 30 min at 37 °C in a humidified chamber. Cells were washed four times for 5 min in Wash Buffer A and the amplification solution was applied for 100 min at 37 °C in a humidified chamber (Duolink In Situ Detection Reagents Green, Sigma, no. DUO92014), then cells were washed three times for 10 min in Wash Buffer B (Sigma, no. DUO82049). Ligation and amplification solutions were prepared per the manufacturer’s instructions. After the PLA protocol, cells were incubated overnight at 4 °C in 5H1 tubulin antibody (1:2000 in 2% GS, RRID: AB_2832941, β-tubulin mouse monoclonal antibody).^42^ Cells were washed three times for 5 min in 1× TBS and incubated in Alexa Fluor 568 goat anti-mouse IgM (1:500 in 2% GS, Thermo Fisher Scientific, no. A-21043, RRID: AB_2535712) for 1 h at room temperature followed by three washes in 1× TBS and DAPI counterstaining.

Cells were imaged using a Nikon A1+ laser scanning confocal microscope equipped with a 60x oil-immersion objective and Nikon Elements AR software. Three to four Z-stack images were collected at 60× magnification for each replicate (ten images/stack with a 0.5 μm step size), and all settings were kept the same between conditions. Z-stack images of PLA puncta and 5H1 tubulin immunofluorescence stains were then analyzed using FIJI.^43^ Maximum intensity projection images were created, and PLA puncta (green channel) were counted using the analyze particles function with a threshold set at a minimum of 400, maximum of 4000, particle size set to a minimum of 0.12 μm^2^, and circularity of 0.00–1.00. Tubulin 5H1 immunofluorescence stain (red channel) was analyzed using the measure function with threshold set at a minimum of 400 and a maximum of 4000 and % area of 5H1 expression was recorded. The number of PLA puncta was normalized to the 5H1 tubulin signal and reported as PLA puncta/% area 5H1.

### Statistical Methods

PLA puncta were analyzed using two-tailed unpaired Student’s *t* test showing statistically significant *p* values as *p ≤ 0.05 and **p ≤ 0.01. The Shapiro-Wilk test was used to assess the normality of the data sets. Data were represented as the mean ± SD. GO cellular component and molecular function showed enriched annotations with FDR ≤ 0.05 and graphed as -Log_10_(FDR). Statistical tests and graphs were created using GraphPad Prism (V. 10.0.2; GraphPad Software, La Jolla California USA).

### Experimental Design and Statistical Rationale

Mass spectrometry identification of biotinylated proteins in BioID2 experiments was performed in three independent biological replicates per experimental or control condition. Experimental conditions included Tau-BioID2 and BioID2-Tau, while the controls were BioID2-HA and Myc-BioID2. Two control proteins were utilized because the position of the 13X linker and the tags used (Myc and HA) were different. For each experimental and control condition, peptides identified by mass spectrometry were filtered by 1% FDR, and at least two peptides were required to identify a protein. For both the qualitative and LFQ analyses, proteins identified in fewer than two experimental replicates and two control replicates were omitted from further analysis. LFQ protein ratios were measured relative to those of the respective control (Tau-BioID2 versus BioID2-HA or BioID2-Tau versus Myc-BioID2). For the LFQ analysis, we used a cutoff abundance ratio of ≥3-fold. It is important to note that we are using the ratios as a cutoff for GO grouping and designating proteins for follow-up validation experiments to confirm interacting partners and, thus, did not consider the LFQ p-values. Validation of tau interacting partners using either co-IP or PLA were performed in three independent biological replicates per condition. Antibody isotype controls were used for the co-IP experiments. PLA experimental controls included omission of each primary antibody and omission of all primary antibodies in MAPT KI neurons as well as the full PLA reaction in TKO neurons. Lists of identified proteins were curated and filtered according to the defined criteria using RStudio (V.1.4.1717).^44^ Schematic figures were created with BioRender.com.

## Results

### Expression of the BioID2 Proteins in Primary Cortical Neuron Cultures

We created lentiviruses that express BioID2-Tau or Tau-BioID2 proteins by fusing the biotin-ligase, BioID2, to either the N-terminus or the C-terminus of the full-length human Tau 2N4R isoform, respectively (Figure 1A). Myc-BioID2 and BioID2-HA were used as controls for N-terminal-tagged BioID2-Tau and C-terminal-tagged Tau-BioID2, respectively (Figure 1A). Immunoblotting of lysates from lentivirus-transduced TKO primary cortical neurons revealed the functional lentiviral expression of the Myc-tagged (BioID2-Tau and Myc-BioID2) and the HA-tagged (Tau-BioID2 and BioID2-HA) proteins at the expected molecular weights (Figure 1B). Immunofluorescence staining showed the presence of biotin labeling of proximal proteins that colocalized with Myc-BioID2, BioID2-Tau, BioID2-HA, and Tau-BioID2 expression in the neuronal processes and somata (Figure 1C,D). Tau was expressed in primary neurons transduced by BioID2-Tau or Tau-BioID2 lentiviruses but not the Myc-BioID2 and BioID2-HA control lentiviruses (Figure 1C,D). Nontransduced primary neurons showed no streptavidin signal which confirmed that the biotinylation signal was due to labeling by BioID2 rather than being endogenously biotinylated (). Together, these data confirm that the lentiviruses were functional, and the expressed BioID2 proteins were capable of *in situ* biotin labeling of the proximal proteins in TKO primary cortical neurons.

**Figure 1:**
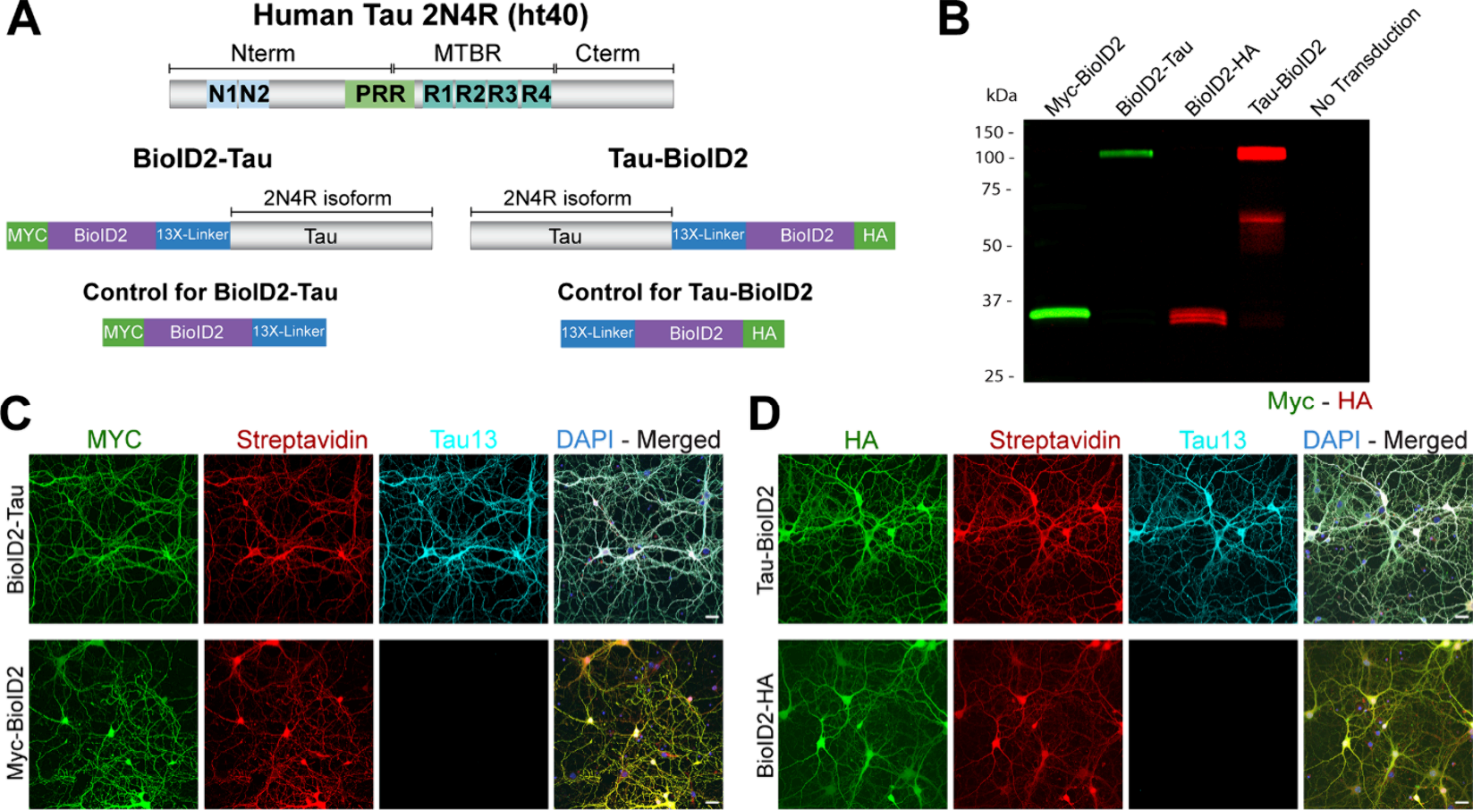
BioID2 proteins expression in primary cortical neurons. *A*, schematic representation of the tau isoform and BioID2 constructs used in this study. BioID2 was fused to either the N-terminus (BioID2-Tau) or the C-terminus (Tau-BioID2) of the human 2N4R tau isoform (ht40). The 2N4R isoform consists of the N-terminal domain (Nterm) with two exon inserts (N1 and N2), the proline rich region (PRR), the microtubule binding region with four repeat domains (MTBR), and the C-terminal domain (Cterm). We generated Myc-BioID2 and BioID2-HA control constructs, which express only the BioID2 protein with its respective tag. BioID2 constructs feature a 13X-linker that increases the biotinylation range and minimizes the interaction between tau and BioID2. *B*, representative immunoblot of lentiviral expression in TKO primary cortical neurons. Lentiviruses expressed Myc-BioID2 at ∼37 kDa (green), BioID2-Tau at ∼100 kDa (green), BioID2-HA at ∼37 kDa (red), and Tau-BioID2 at ∼100 kDa (red). No expression of the BioID2 proteins was observed in the nontransduced cells (full representative blot in ). *C*, immunofluorescence showing the lentiviral expression of the BioID2-Tau and Myc-BioID2 proteins (Myc, green) and biotinylation of proximal proteins (streptavidin, red) in a TKO primary cortical neuron culture. Tau was only expressed in BioID2-Tau (Tau13, cyan) but not in the control Myc-BioID2 transduced culture. *D*, immunofluorescence showing the lentiviral expression of the Tau-BioID2 and BioID2-HA proteins (HA, green) and biotinylation of proximal proteins (streptavidin, red) in a TKO primary cortical neuron culture. Tau was only expressed in Tau-BioID2 (Tau13, cyan) but not in the control BioID2-HA transduced culture. Scale bars are 25 μm. N1–N2, N-terminal inserts; PRR, proline rich region; R1–R4, repeat domains of the MTBR.

### Mass Spectrometry Identification of Biotinylated Proteins

We utilized the BioID2 *in situ* labeling approach to identify candidate tau protein–protein interactions in primary cortical neurons expressing BioID2 tau and control proteins. The biotinylated proteins were pulled down using streptavidin-coated magnetic beads, digested, and then identified using mass spectrometry (Figure 2A). Immunoblotting revealed the successful elution of the biotinylated proteins from the streptavidin beads (Figure 2B). Distinct band patterns suggest differences in proteins interacting with the tau fusion constructs (Tau-BioID2 and BioID2-Tau) compared to their respective controls (BioID2-HA and Myc-BioID2).

**Figure 2:**
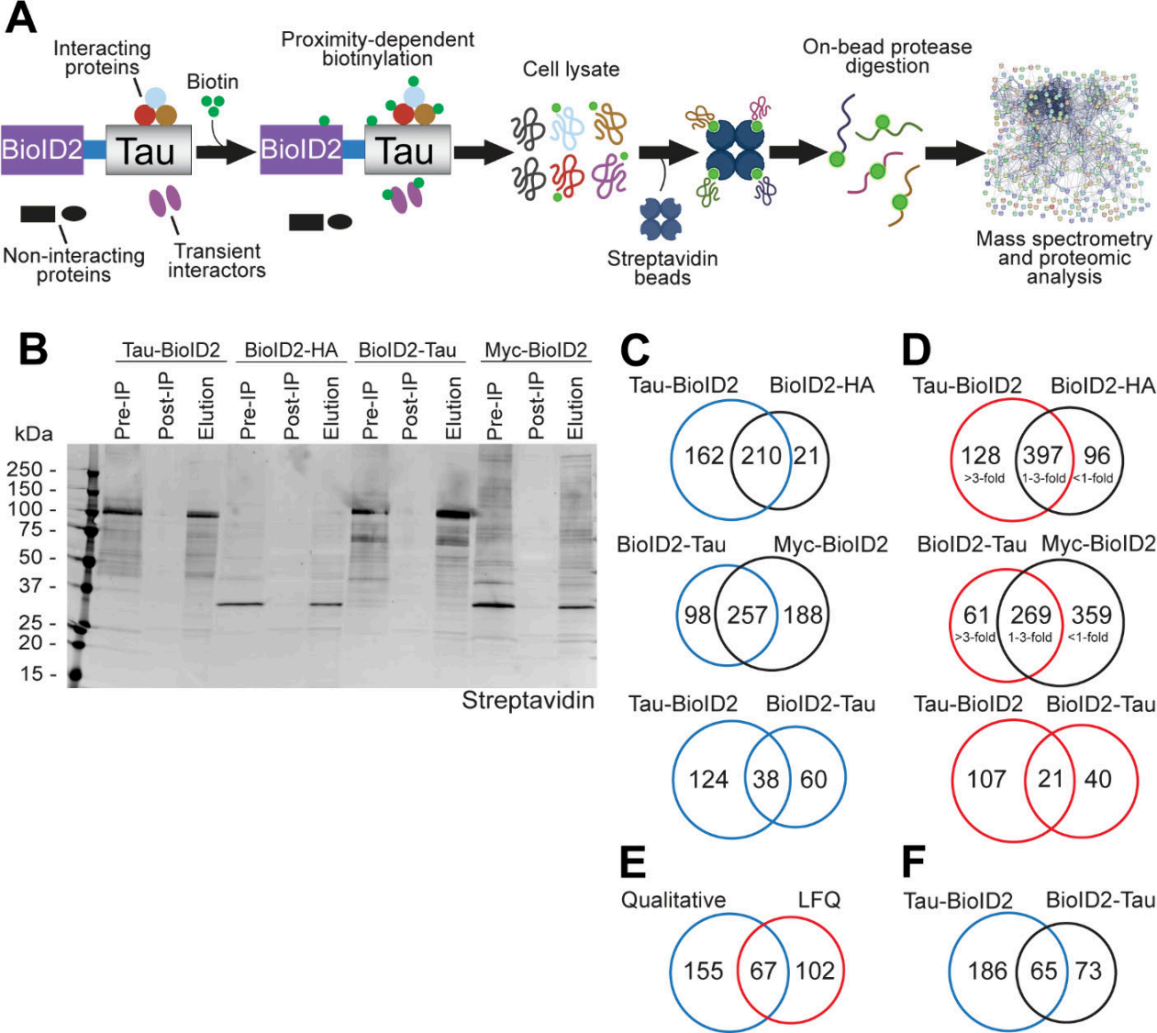
Mass spectrometry identification of biotinylated proteins. *A*, schematic representation of the BioID2 approach for *in situ* labeling of proximal proteins. Fusion proteins were created between Tau and BioID2. In neurons, proteins either form stable complexes with tau or interact transiently with tau. BioID2 only labels proteins that are in close proximity (within a labeling radius of ∼10 nm) in living neurons, and the labeled proteins can accumulate over time. The biotinylated proteins were then affinity-captured using streptavidin magnetic beads and identified by mass spectrometry. *B*, immunoblotting with the streptavidin antibody shows successful capturing and elution of biotinylated proteins. Lysates were collected from TKO primary cortical neurons transduced with Tau-BioID2, BioID2-HA, BioID2-Tau, or Myc-BioID2 (*n* = 3 biological replicates per experimental condition, full blot is shown in B). *C*, qualitative analysis of the mass spectrometry data identified 162 proteins as potential interactors with Tau-BioID2 compared to the BioID2-HA control and 98 proteins with BioID2-Tau compared to the Myc-BioID2 control. *D*, LFQ identified 128 proteins as potential Tau-BioID2 interactors compared to the BioID2-HA control and 61 with BioID2-Tau when compared to the Myc-BioID2 control. *E*, in combining the qualitative and LFQ analyses, 155 proteins were identified using the qualitative analysis, 102 with the LFQ analysis, and 67 proteins were identified in both approaches. *F*, Venn diagram showing the number of proteins identified with Tau-BioID2 only (186 proteins), BioID2-Tau only (73 proteins), or both fusion proteins (65 proteins). LFQ, label-free quantification.

First, proteins were qualitatively identified from the Tau-BioID2, BioID2-Tau, BioID2-HA, and Myc-BioID2 samples. We included proteins that were identified in at least two of the three experimental replicates. Utilizing this approach, we identified 162 proteins in the C-terminal-tagged Tau-BioID2 that were not present in the BioID2-HA control. There were 210 proteins shared between the Tau-BioID2 and BioID2-HA control samples, which were not considered further (Figure 2C upper panel and ). We identified 98 proteins with BioID2-Tau that were not identified in the Myc-BioID2 control samples. There were 257 proteins shared between the BioID2-Tau and Myc-BioID2 control, which were not considered further (Figure 2C middle panel and ). Among the unique Tau-BioID2 and BioID2-Tau interactors, 38 proteins were shared between the tau proteins, 124 were identified only in Tau-BioID2 and 60 were identified only in BioID2-Tau, totaling 222 potential tau interacting proteins (Figure 2C lower panel and ). Lists of peptides identified from the qualitative analysis are found in . For a more stringent analysis, we considered only proteins identified in all three replicates of each experimental sample. This analysis resulted in 99 candidate interactors for Tau-BioID2 and 41 for BioID2-Tau (). Comparing the two methods, 144 proteins were unique to the two-out-of-three replicates analysis, 43 proteins were unique to the three-out-of-three replicates analysis, and 78 proteins were common in both analyses ().

Second, we performed LFQ analysis to quantify the protein abundance ratios of the identified proteins. Each experimental condition, Tau-BioID2 or BioID2-Tau, was compared to that of its respective control BioID2-HA or Myc-BioID2. We excluded proteins that were identified in only one replicate and applied a threshold of ≥3-fold increase compared with the respective control. We identified 128 proteins as potential interactors with Tau-BioID2 compared to the BioID2-HA control and 61 proteins as potential interactors with BioID2-Tau compared to the Myc-BioID2 control (Figure 2D upper and middle panels and ). Among the proteins identified by the LFQ method, 21 proteins were shared between Tau-BioID2 and BioID2-Tau, 107 were identified only in Tau-BioID2 and 40 were identified only in BioID2-Tau, totaling 168 potential tau protein interactors (Figure 2D lower panel and ). Lists of peptides identified from the LFQ analysis are listed in .

Next, we compiled the proteins identified by qualitative and quantitative analyses, which included a total of 324 proteins. Among these proteins, 155 were identified using the qualitative method, 102 were identified using LFQ analysis, and 67 proteins were detected in both analyses (Figure 2E and ). Among the 324 proteins, 186 proteins were identified with only Tau-BioID2, 73 proteins were identified with only BioID2-Tau, and 65 proteins were identified with both tau proteins (Figure 2F and ).

GO cellular component enrichment analysis indicated that the identified proteins were associated with various cellular compartments, including the cytoplasm (296 proteins), mitochondria (118 proteins), ribonucleoprotein complex (58 proteins), synapses (79 proteins), axon (48 proteins), cell body (48 proteins), cytoskeleton (80 proteins), dendrites (43 proteins), and the nucleus (150 proteins) (Figure 3A and ). GO molecular function analysis identified ribosomal proteins (31 proteins), RNA binding proteins (66 proteins), cytoskeletal binding proteins (53 microtubule- and actin-binding proteins), kinase binding proteins (41 proteins), proteins involved in mitochondrial oxidoreductase (33 proteins) and electron transfer activities (6 proteins), proteins involved in translation regulation (13 proteins), chaperone proteins (7 proteins), and ubiquitin ligase binding proteins (15 proteins) (Figure 3B and ). The full lists of the identified proteins in the GO cellular component, GO molecular function, and GO biological process annotations are found in .

**Figure 3:**
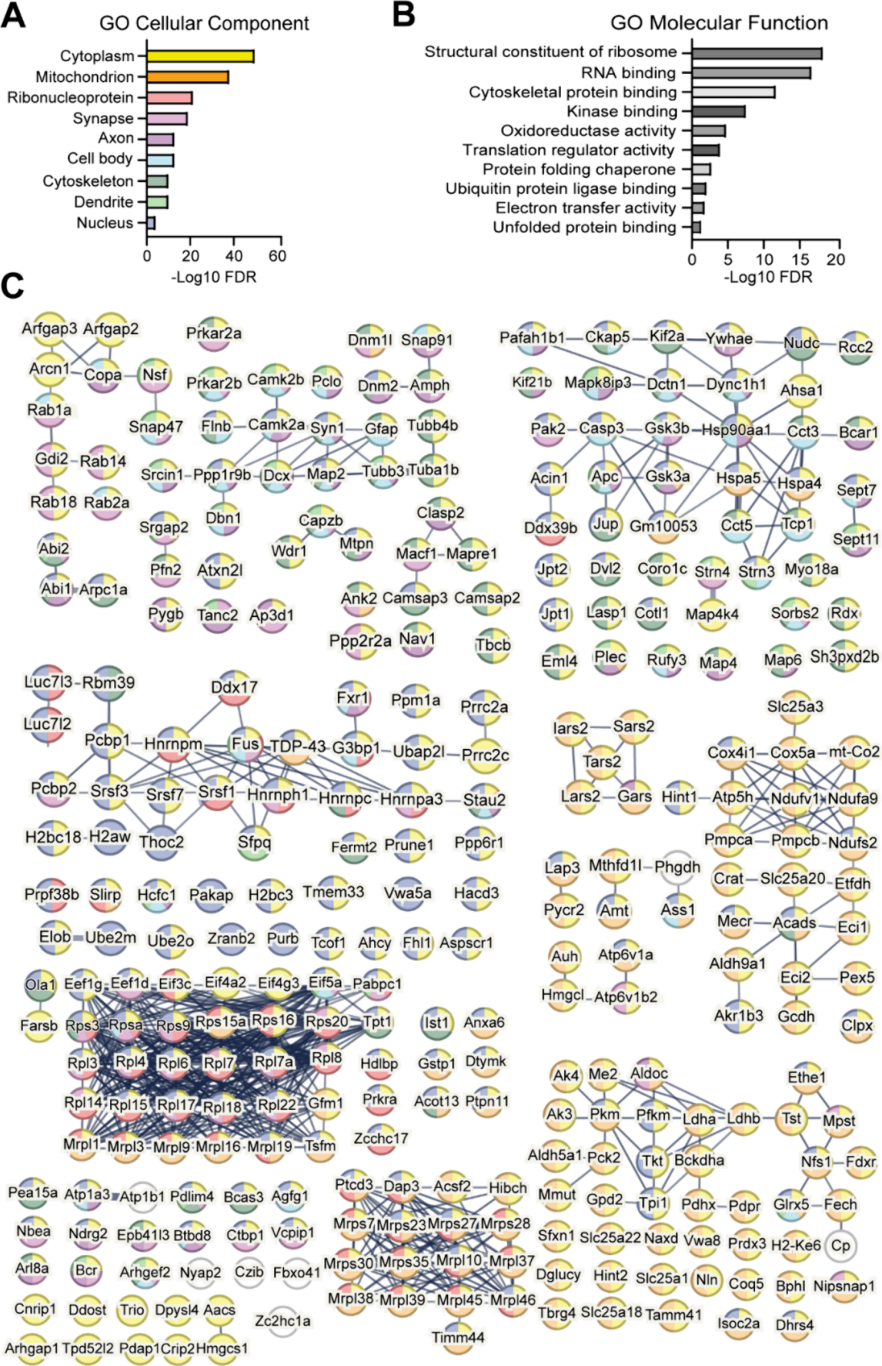
Gene ontology enrichment and STRING analyses of the identified proteins. *A*, GO cellular component analysis includes annotations with a false discovery rate of ≤0.05. Selected annotations were graphed with −log_10_(FDR). The full list of annotations is in . *B*, GO molecular function included annotations with an FDR of ≤ 0.05. Selected annotations were graphed with −log_10_(FDR). The full list of annotations is in . *C*, STRING analysis was used to visualize the protein interactome network, including both functional and physical associations with a minimum required confidence interaction score of 0.7 and an MCL inflation value of 3.0. Nodes were colored based on the following GO cellular components: cytoplasm (yellow), mitochondria (orange), ribonucleoprotein complex (red), synapse (pink), axon (dark pink), cell body (light blue), cytoskeleton (dark green), dendrite (green), and the nucleus (blue). GO, gene ontology; FDR, false discovery rate.

Interestingly, the KEGG pathway analysis identified 26 proteins associated with human diseases. Proteins associated with AD included GSK3β, CASP3, DVL2, and APC, among others. Proteins associated with Parkinson’s disease included HSPA5, and CAMK2A, among others. Proteins associated with amyotrophic lateral sclerosis included TDP-43, FUS, and SRSF3, among others. Proteins associated with Huntington’s disease included CASP3, Cox5a, and Cox4i1, among others. The full list of the identified proteins in the KEGG pathway analysis is found in .

Functional and physical association networks of the identified tau interacting partners (324 proteins) were created using STRING with an MCL inflation value of 3.0 (Figure 3C). GO cellular component analysis for the proteins included in the three-out-of-three replicates analysis identified proteins associated with cytoplasm (110 proteins), mitochondria (44 proteins), synapses (38 proteins), cell body (24 proteins), axon (22 proteins), dendrite (20 proteins), cytoskeleton (33 proteins), and ribonucleoproteins (16 proteins) (). The identified GO cellular components were similar to those reported in the two-out-of-three analysis. Full lists of proteins in the GO cellular component and GO molecular function annotations are provided in .

We compared our list of 324 potential tau interacting proteins to a list of tau interactors published in a comprehensive review article that included proteins identified in seven tau interactome studies using human tissue or human cell models (including the APEX2-based proximity labeling tau interactome study) as well as five tau interactome studies in mouse models comprising a total of >3700 proteins.^9^ Interestingly, 247 proteins identified here were also identified in at least one study of the 12 studies included in the review, while 77 proteins were uniquely identified in our study (). We also compared our list to three proximity-dependent biotinylation studies.^27–29^ Tracy et al. fused APEX2 to the N-terminus or the C-terminus of human tau (2N4R) and expressed the fusion constructs in stem cell-derived i^3^Neurons. There were 50 proteins shared between our work and the APEX2 study, while 274 proteins were unique to the BioID2 approach (). Two recently published tau interactome studies utilized the BioID2 approach in various models and were not included in the Kavanagh et al. review.^28,29^ Prikas and colleagues fused BioID2 to the N-terminus of full-length human tau (2N4R isoform) and expressed the fusion protein in wild-type mouse primary cortical neurons, in the cortex and hippocampus of P35 wild-type mice, and in the hippocampus of six-month old TKO mice.^28^ From the 324 proteins identified in our study, 27 were shared with the wild-type mouse primary neuron list, 24 were shared with the P35 mouse cortex and hippocampus protein list, and 46 were shared with the hippocampal TKO mouse list (). Thus, nearly 300 proteins were uniquely identified in our study compared to the Prikas et al. study (). Griffin and colleagues fused BioID2 to the C-terminus of the 0N4R tau isoform and identified tau protein interactions in fibril (K18)-treated human embryonic stem cells-derived NGN2 neurons.^29^ Our tau protein interactome list included 16 shared proteins and 308 unique proteins (). The criteria used to define candidate tau interactors in the previously published tau interactome studies are summarized in .

### BioID2 Tau Identified Cytoskeletal Proteins as Potential Interactors

GO cellular component analysis identified 80 proteins associated with the cytoskeleton (Figure 4A). The identified cytoskeletal proteins included tubulin and tubulin binding proteins (e.g., MAP2, MAP6, MAPRE1, KIF2A, KIF21B, DNM2, DCTN1 CAMSAP2, and CAMPSAP3), actin binding proteins (e.g., DBN1, CAPZB, ARPC1A, FLNB, and PFN2), and kinases or kinase-binding proteins (e.g., GSK3β, GSK3α, CAMK2B, PRKAR2A, and PRKAR2B) (Figure 4A,B and ).

**Figure 4:**
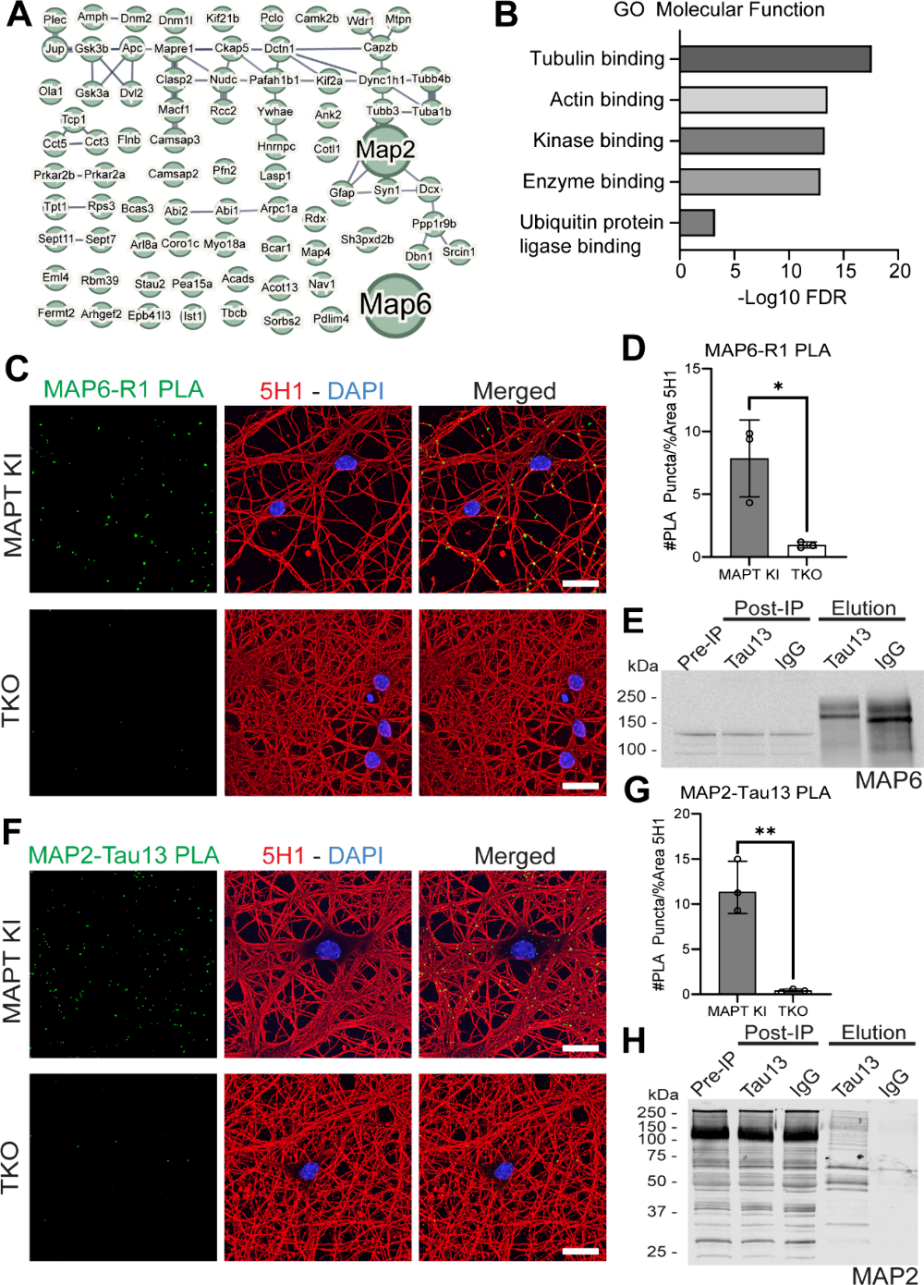
Validation of potential interactions between tau and other cytoskeletal proteins. *A*, STRING network mapping the functional and physical associations of the proteins identified as candidate tau interactors associated with the cytoskeleton. MAP2 and MAP6 are denoted as large nodes to indicate inclusion in the validation experiments. *B*, selected GO molecular function annotations related to the identified cytoskeleton proteins (FDR ≤ 0.05). The full list of annotations is in . *C*, the association between tau and MAP6 was validated using MAP6 and R1 antibody PLA. Many PLA puncta (green) are seen in MAPT KI cortical neurons (top panels) when compared with TKO cortical neurons (bottom panels). Tubulin immunofluorescence staining was performed using 5H1 antibody (red) and a nuclear stain was performed using DAPI (blue). Scale bars are 20 μm. *D*, quantification of the MAP6-R1 PLA (*n* = 3 biological replicates) shows significantly greater PLA signal in MAPT KI neurons when compared to TKO neuron controls. *E*, co-IP using Tau13 antibody (*n* = 3 biological replicates) showed no MAP6 bands in the elution lanes. Note that bands present in the elution are from the IP IgG antibodies (full representative blot shown in ). *F*, the association between tau and MAP2 was validated using MAP2 and Tau13 antibody PLA. Many PLA puncta (green) are seen in MAPT KI cortical neurons (top panels) when compared to TKO cortical neurons (bottom panels). Tubulin immunofluorescence staining was performed using 5H1 antibody (red) and a nuclear stain was performed using DAPI (blue). Scale bars are 20 μm. *G*, quantification of the MAP2-Tau13 PLA (*n* = 3 biological replicates) shows significantly greater PLA signal in MAPT KI neurons compared to TKO neuron controls. *H*, co-IP using Tau13 antibody (*n* = 3 biological replicates) showed MAP2 bands in the Tau13 elution lane, with low signal in IgG control elution lane (full representative blot shown in ). Unpaired Student’s *t* test was used for PLA quantification *p ≤ 0.05 and **p ≤ 0.01. Pre-IP indicates the lysate; post-IP indicates the supernatant; PLA, proximity ligation assay; co-IP, coimmunoprecipitation; MAPT KI, human MAPT knock-in; TKO, tau knockout; FDR, false discovery rate.

We utilized two different approaches to validate tau protein–protein interactions and associations with cytoskeletal proteins: co-IP from human MAPT KI mouse cortices and PLA performed on MAPT KI primary cortical neurons. MAP6 was identified as a candidate tau protein interactor in the qualitative and LFQ analyses (). In the qualitative analysis, MAP6 was identified as a candidate interactor in both the two-out-of-three and three-out-of-three analyses (). PLA using MAP6 and tau (R1) antibodies showed significantly more PLA puncta in MAPT KI cortical neurons compared to the TKO cortical neuron controls (Figure 4C,D). Additional controls included performing PLA in MAPT KI cortical neurons with the R1 antibody omitted (R1^–^/MAP6^+^; ), MAP6 antibody omitted (R1^+^/MAP6^–^; ), and both antibodies omitted (R1^–^/MAP6^–^; ), all of which produced low PLA signal. We also performed traditional protein–protein interaction co-IP assays. Using the Tau13 antibody to purify tau, we did not detect neuronal MAP6 (∼120 kDa) or oligodendrocytic MAP6 (∼90 kDa) in the elution lanes (note bands present in the elution are from the IP antibodies; Figure 4E). A nonspecific IgG isotype antibody was used as a control.

MAP2 was identified as a candidate tau protein interactor in the LFQ analysis only (). The PLA reaction with MAP2 and Tau13 antibodies showed significantly increased PLA puncta in MAPT KI cortical neurons compared to TKO neuron controls (Figure 4F and G). Additional controls included performing PLA in MAPT KI cortical neurons with the Tau13 antibody omitted (Tau13^–^/MAP2^+^; ), MAP2 antibody omitted (Tau13^+^/MAP2^–^; ) and both antibodies omitted (Tau13^–^/MAP2^–^; ), all of which produced low PLA signal. Co-IP using Tau13 antibody and IgG isotype antibody as a control showed MAP2 bands present in the Tau13 elution lane, with low signal in the IgG control lane (Figure 4H).

### BioID2 Tau Identified Nuclear and Ribonucleoprotein Complex Proteins as Potential Interactors

The identified proteins in our studies included 150 proteins associated with the nucleus or the ribonucleoprotein complex (truncated network in Figure 5A, full list in ). GO molecular function identified nuclear and nucleic acid binding proteins (e.g., FUS, TDP-43, SRSF7, SRSF1, SRSF3, prune1, and DDX17), ribosomal proteins (e.g., MRPL10, MRPS23, RPL6, and RPS3), and ribonucleoproteins (e.g., HNRNPA3, HNRNPC, HNRNPH1, and HNRNPM) (Figure 5A,B and ).

**Figure 5:**
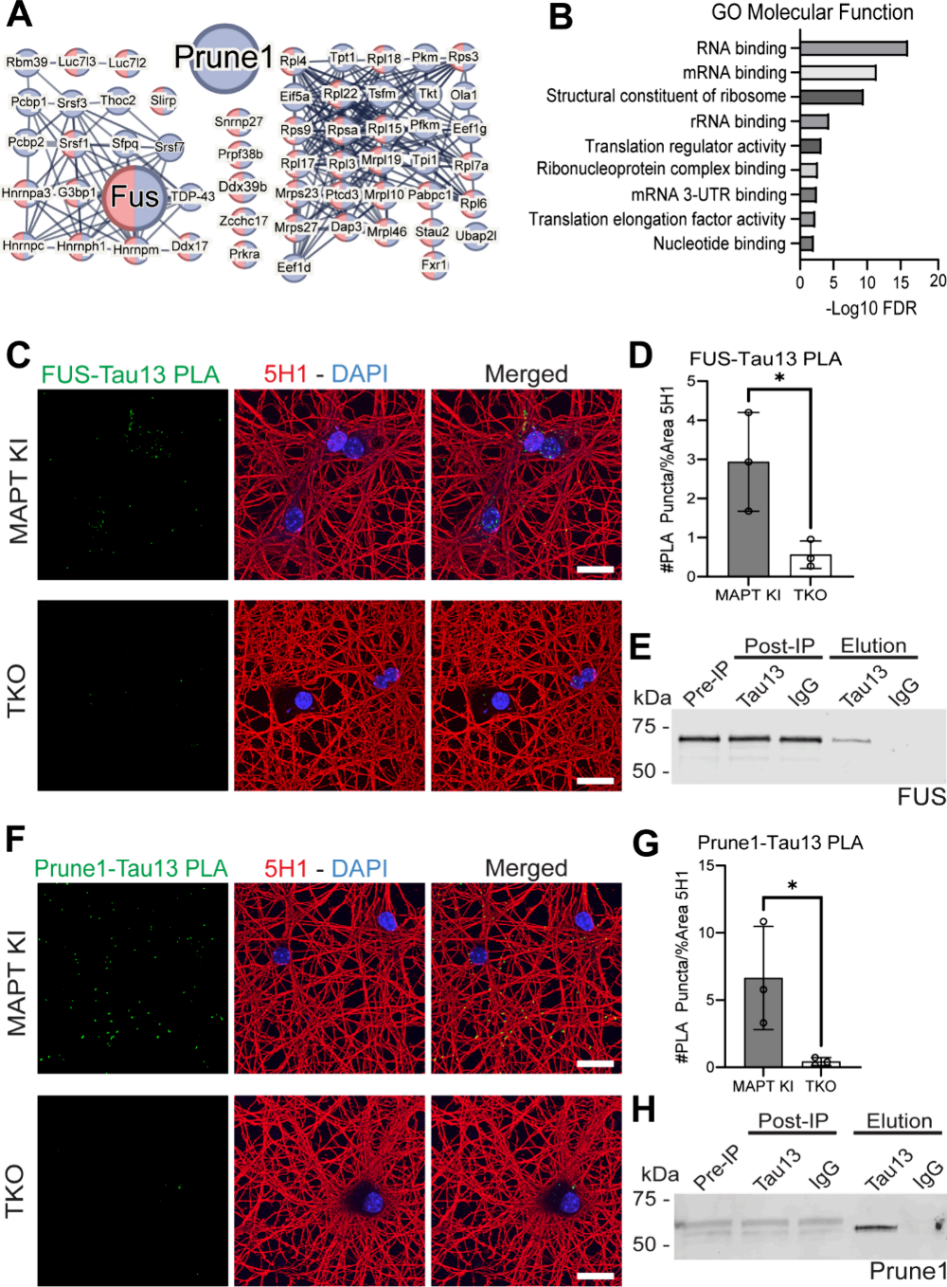
Validation of potential interactions between tau and proteins associated with the nucleus and ribonucleoprotein complex. *A*, STRING network mapping the functional and physical associations of candidate tau interactors related to the nucleus or the ribonucleoprotein complex. FUS and prune1 are denoted as large nodes to indicate their inclusion in validation experiments. *B*, selected GO molecular function annotations of candidate tau interactors related to the nucleus and the ribonucleoprotein complex. The full list of annotations is in . *C*, the association between tau and FUS was validated using FUS and Tau13 antibody PLA. Many PLA puncta (green) are seen in MAPT KI cortical neurons (top panels) when compared to TKO cortical neurons (bottom panels). Tubulin immunofluorescence staining was performed using 5H1 antibody (red) and a nuclear stain was performed using DAPI (blue). Scale bars are 20 μm. *D*, quantification of the FUS-Tau13 PLA (*n* = 3 biological replicates) shows significantly greater PLA signal in MAPT KI neurons when compared to TKO neuron controls. *E*, co-IP using Tau13 antibody (*n* = 3 biological replicates) showed a FUS band in Tau13 elution lane but not in IgG control lane (full representative blot shown in ). *F*, the association between tau and prune1 was validated using prune1 and Tau13 antibody PLA. Many PLA puncta (green) are seen in MAPT KI cortical neurons (top panels) when compared to TKO cortical neurons (bottom panels). Tubulin immunofluorescence staining was performed using 5H1 antibody (red) and a nuclear stain was performed using DAPI (blue). Scale bars are 20 μm. *G*, quantification of the prune1-Tau13 PLA (*n* = 3 biological replicates) shows significantly greater PLA signal in MAPT KI neurons compared to TKO neuron controls. *H*, co-IP using Tau13 antibody (*n* = 3 biological replicates) showed a prune1 band in the Tau13 elution lane but not in IgG control elution lane (full representative blot shown in ). Unpaired Student’s *t* test was used for PLA quantification *p ≤ 0.05 and **p ≤ 0.01. Pre-IP indicates the lysate; post-IP indicates the supernatant; PLA, proximity ligation assay; co-IP, coimmunoprecipitation; MAPT KI, human MAPT knock-in; TKO, mouse tau knockout; FDR, false discovery rate.

FUS was identified as a candidate tau protein interactor in the LFQ analysis only. We validated the interaction/association of endogenous tau with FUS by using both co-IP and PLA. FUS-Tau13 PLA signal was significantly increased in MAPT KI neurons when compared to TKO neuron controls (Figure 5C,D). The primary antibody delete controls showed a low number of PLA puncta in MAPT KI neurons (). Moreover, FUS co-IPed with tau from human MAPT KI cortical tissue lysates shows an ∼70 kDa band present in the Tau13 elution lane but not in the IgG isotype control elution lane (Figure 5E).

Prune1 was identified in qualitative analysis only. Prune1 was included in both the two-out-of-three and the three-out-of-three analyses (). We validated the interaction/association of endogenous tau with prune1 using both co-IP and PLA. Prune1-Tau13 PLA showed a significantly increased signal in MAPT KI neurons compared to TKO neurons (Figure 5F,G). The primary antibodies delete controls showed a low number of PLA puncta in MAPT KI neurons (). Finally, co-IP from MAPT KI cortical tissue lysates showed an ∼60 kDa band present in the Tau13 elution lane but not in the IgG isotype control lane (Figure 5H).

### BioID2 Tau Identified Synaptic Proteins as Potential Interactors

GO cellular component analysis identified 79 proteins associated with the synapses (truncated network in Figure 6A, full list in ), including presynaptic proteins (e.g., PCLO, BTBD8, APC, and RAB1A), synaptic vesicle proteins (e.g., synapsin-1, SNAP47, SNAP91, AMPH, RAB2A, and RAB14), and postsynaptic proteins (e.g., neurabin-2, TANC2, and septin-11) (Figure 6B and ).

**Figure 6:**
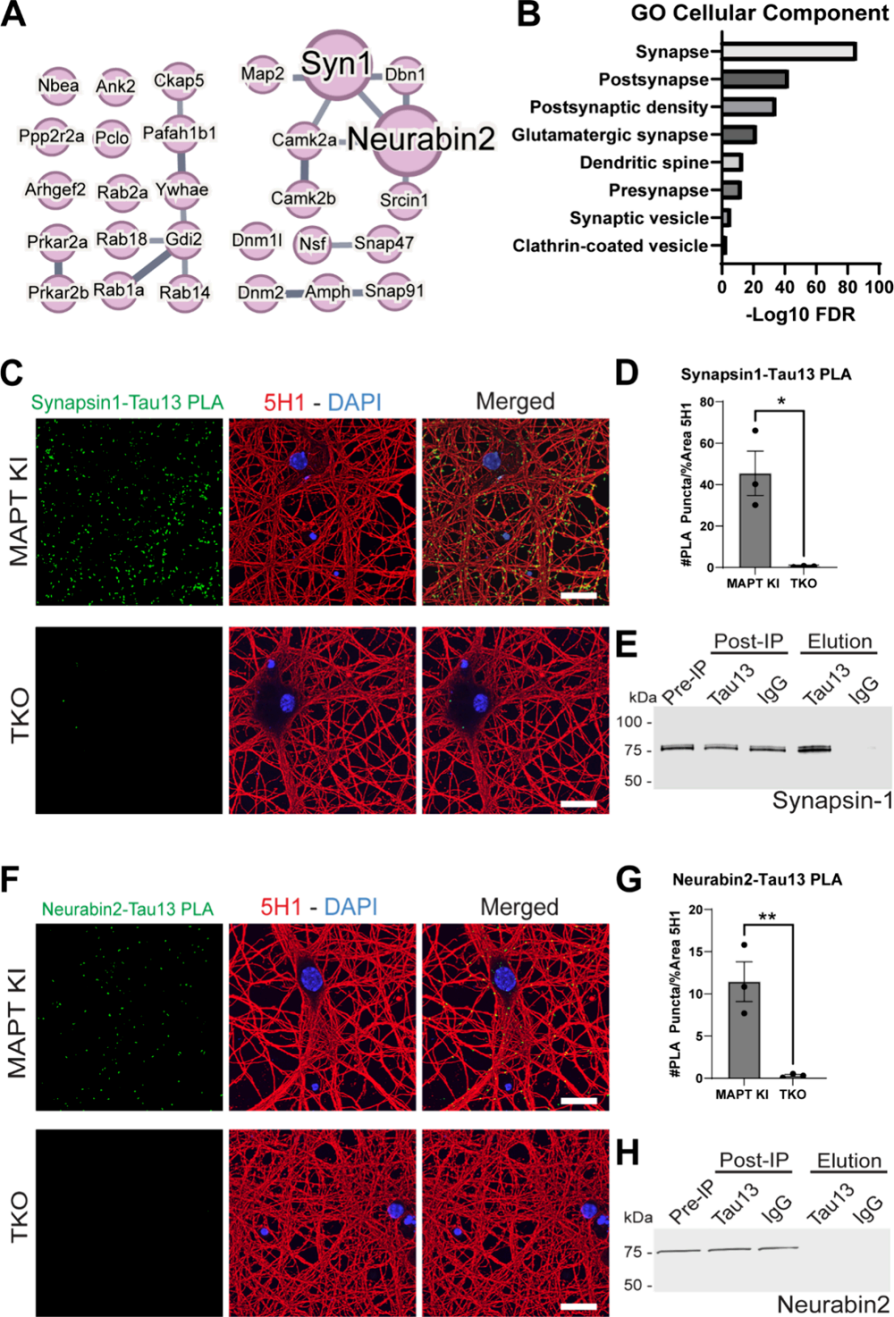
Tau interactome mapping using the BioID2 approach identified proteins associated with synapses. *A*, STRING network mapping of functional and physical associations of candidate tau interactors identified proteins associated with synapses. Synapsin-1 and neurabin-2 are denoted as large nodes to indicate inclusion in the validation experiments. *B*, selected GO cellular component analysis of candidate tau interactors identified proteins associated with synapses. The full list of annotations is in . *C*, the association between tau and synapsin-1 was validated via PLA using synapsin-1 and Tau13 antibodies. Many more PLA puncta (green) are present in MAPT KI cortical neurons (top panels) when compared with TKO cortical neurons (bottom panels). Tubulin immunofluorescence staining was performed using 5H1 antibody (red) and a nuclear stain was performed using DAPI (blue). Scale bars are 20 μm. *D*, quantification of the synapsin-1-Tau13 PLA (*n* = 3 biological replicates) shows significantly greater PLA signal in MAPT KI neurons when compared to TKO neuron controls. *E*, synapsin-1 copurified with tau in a co-IP using the Tau13 antibody (*n* = 3 biological replicates) but was not present in the IgG control elution (full representative blot shown in ). *F*, the association between tau and neurabin-2 was validated using neurabin-2 and Tau13 antibody PLA. More PLA puncta (green) are observed in MAPT KI cortical neurons (top panels) than in the TKO cortical neurons (bottom panels). Tubulin immunofluorescence staining was performed using 5H1 antibody (red) and a nuclear stainwas performed using DAPI (blue). Scale bar 20 μm. *G*, quantification of the neurabin-2 and Tau13 PLA (*n* = 3 biological replicates) shows significantly greater PLA signal in MAPT KI neurons compared to TKO neuron controls. *H*, co-IP using Tau13 antibody (*n* = 3 biological replicates) showed no neurabin-2 bands in the elution lanes (full representative blot shown in ). Unpaired Student’s *t* test was used for PLA quantification *p ≤ 0.05 and **p ≤ 0.01. Pre-IP indicates the lysate, and post-IP indicates the supernatant. PLA; proximity ligation assay, co-IP; coimmunoprecipitation, MAPT KI; human MAPT knock-in, TKO; mouse tau knockout, FDR; false discovery rate.

Synapsin-1 was identified as a tau candidate protein interactor by both the qualitative and LFQ analyses. In the qualitative analysis, synapsin-1 was included in the two-out-of-three analysis, but not in the three-out-of-three analysis (). We validated the interaction/association between endogenous human tau and synapsin-1 using co-IP and PLA. Synapsin-1-Tau13 PLA showed significantly more PLA puncta in MAPT KI neurons compared with TKO neurons (Figure 6C and D). Primary antibody deletes showed little to no PLA puncta in MAPT KI neurons (). Synapsin-1 also co-IPed with endogenous human tau from cortical tissue lysates, showing an ∼77 kDa band in the Tau13 elution lane but not in the IgG isotype control lane (Figure 6E). The synapsin-1 antibody detected both isoforms of synapsin-1 (synapsin-1a and synapsin-1b) shown as two bands co-IPed with tau (Figure 6E).

Neurabin-2 was identified by a qualitative method only and was included in both the two-out-of-three and the three-out-of-three analyses (). The interaction/association between human tau and neurabin-2 was validated using co-IP and PLA. The Neurabin-2-Tau13 PLA signal was significantly higher in MAPT KI neurons than in TKO neurons (Figure 6F,G). Primary antibody deletes showed little to no PLA puncta in MAPT KI neurons (). A co-IP using the Tau13 antibody showed no neurabin-2 bands in the elution lanes of Tau13 or the IgG isotype control (Figure 6H).

## Discussion

Tau is a key protein involved in regulating a multitude of microtubule-dependent and -independent neuronal functions in multiple subcellular compartments.^2–5^ Tau interactome studies in human post-mortem tissue, human cell lines, or animal models identified >2000 proteins as potential tau interacting partners,^9^ suggesting that protein–protein interactions may mediate the diverse cellular functions of tau. In this study, we utilized the BioID2-based proximity-dependent biotinylation approach to study tau-protein interactions *in situ* within neurons. We leveraged the ability of BioID2 to accumulate labeling of direct, weak, and transient protein–protein interactions within close proximity to tau (≤10 nm) and complemented the BioID2 approach with exploratory mass spectrometry. We identified 324 proteins as candidate members of the tau protein interactome which included proteins associated with multiple cellular compartments such as the cytoskeleton, mitochondria, synapses, ribonucleoprotein complex, and the nucleus. Selected proteins were validated with two independent protein–protein interaction/association assays: co-IP and PLA. In the qualitative mass spectrometry analysis, we performed two-out-of-three and three-out-of-three replicate analyses. Synapsin-1 was included in the two-out-of-three analysis but not in the three-out-of-three analysis. The interaction between tau and synapsin-1 was validated using two independent approaches (co-IP and PLA), suggesting that applying a highly stringent analysis may increase the risk of false negatives, leading to the exclusion of true tau protein interactors. MAP6, prune1, and neurabin-2 were included in both analyses and their interaction with tau was validated by either co-IP, PLA, or both suggesting that these proteins could be *bona fide* tau interactors.

We identified 80 cytoskeleton-associated proteins as part of the tau interactome, consistent with previous tau interactome studies (reviewed in^9^) as well as tau’s function as a microtubule-associated protein.^45^ Through three independent approaches (i.e., BioID2/mass spectrometry, co-IP, and PLA), we identified tau-MAP2 protein–protein interactions using 2N4R human tau, supporting prior work. Tau interaction with MAP2 was identified in previous tau interactome studies in human AD post-mortem brain tissue,^10,11^ and abnormally phosphorylated tau isolated from AD brain was reported to interact with MAP2 *in vitro*.^46^ The biological function of this interaction remains unknown but may be related to regulating microtubule dynamics in the somatodendritic compartment of neurons, where both proteins are found. We identified MAP6 as a potential tau interactor using the BioID2/mass spectrometry approach, which was validated using PLA. In this case, the traditional co-IP did not reveal a tau-MAP6 relationship, highlighting the utility of the *in situ* protein labeling and PLA approaches (neither require cell lysis). One possible explanation is that MAP6 could be a transient tau interactor that is lost during the co-IP procedure. Notably, the interaction between tau and MAP6 was also reported in other *in situ* enzyme-mediated proximity labeling studies.^27,28^ This interaction may be important for regulating the labile and stable domains of the microtubules in axons.^47^

Tau’s interactions with RNA-binding proteins and members of the ribonucleoprotein complex are reported extensively in the literature.^9,19,48^ This is consistent with our finding of 150 candidate tau interactors associated with the nucleus or the ribonucleoprotein complex. FUS is an RNA-binding protein that regulates the alternative splicing of exon 10 of the *MAPT* gene.^49,50^ FUS was previously reported as a tau interactor in human neuroblastoma SH-SY5Y cells expressing the 2N4R tau isoform^16^ and in human IMR-32 and ReN VM cell lines expressing 2N4R and 2N3R tau isoforms.^15^ However, to our knowledge, this is the first study providing evidence that FUS interacts with 2N4R human tau in primary neurons and brain tissue using three different approaches (i.e., BioID2/mass spectrometry, co-IP, and PLA). The importance of the tau-FUS interaction remains unknown.

In this study, we reported 79 proteins associated with the synapses as candidate members of the tau interactome. This is consistent with previous tau interactome studies, which identified synaptic proteins as tau interactors.^9,27^ The interaction of tau and synapsin-1 was reported in post-mortem human brain tissue,^11,13^ in the transgenic SHR72 rat model,^18^ and with endogenous mouse tau in brain tissue.^20^ We validated the interaction of tau and synapsin-1 with both co-IP and PLA. Synapsin-1 is a phosphoprotein that binds to and regulates dynamics of synaptic vesicles.^51,52^ The function and regulators of the interaction between 2N4R human tau and synapsin-1 in synapses requires further investigation, but tau may localize to neuronal synapses.^53–58^ We validated the interaction between tau and neurabin-2 using PLA, but neurabin-2 did not co-IP with tau. Neurabin-2 is a scaffold protein that regulates signal transduction pathways,^59^ much like tau,^4,60^ and it is believed to target protein phosphatase 1 (PP1) to dendritic spines.^59^ Moreover, PP1α was identified as tau interactor in this study, and we previously reported an interaction between tau and PP1 isoforms (α and γ), with tau regulating axonal transport through PP1γ.^4,61–63^ Whether there is a role of neurabin-2 in the tau-PP1 functional interaction remains to be identified.

We compared our list of candidate tau interactors to published lists of tau interactome studies that included antibody-based co-IP approaches^9–16,18,20,27,48^ comprehensively reviewed in.^9^ Interestingly, 76% (247/324) of proteins identified in this study were identified in at least one study reviewed in,^9^ while 77 proteins were unique to this study. Five of the proteins validated (MAP6, MAP2, FUS, synapsin-1, and neurabin-2) were among the 247 proteins identified in other tau interactome studies, while prune1 was among the 77 proteins that were unique to the BioID2 approach in this study. We validated tau-prune1 interaction using both co-IP and PLA. Prune1 is an exopolyphosphatase that interacts with GSK3β (a tau kinase also identified in this work),^64^ co-IPs with β-tubulin (a well-known tau interactor) in neuronal SHSY5Y cells, and enhances microtubule polymerization,^65^ suggesting a role for prune1 in tau-mediated microtubule-dependent functions. It is worth noting that the criteria used to define tau protein candidate interactors vary among the previously published studies, which could influence the differences among the various tau interactome lists.

We evaluated the proteomic data through qualitative and quantitative LFQ analyses to broaden our list of candidate tau interacting proteins that could be confirmed by validation experiments. Proteins validated as tau interactors in this study were either identified by the qualitative comparison of samples (prune1 and neurabin-2), label-free quantification (MAP2 and FUS), or both (MAP6 and synapsin-1) suggesting that analyzing mass spectrometry RAW data using both approaches is beneficial in identifying potential protein–protein interactions. All six protein interactions were successfully validated by PLA, confirming at least a close association between the proteins (<40 nm) and tau. However, we failed to detect MAP6 and neurabin-2 interactions using co-IP. This could be due to the interactions being weak and/or transient, or due to the technical conditions that can impact IP success (temperature, lysis buffer, antibody, etc.). This highlights the utility of validating protein–protein interactions with independent complementary approaches and including techniques that do not require cell disruption.

Furthermore, we compared the list of candidate tau interactors to three studies that utilized proximity-dependent biotinylation to identify tau interactors.^27–29^ Our approach identified 274 proteins that were not identified by the APEX2 approach, while 50 proteins overlapped between both approaches.^27^ This could be explained in terms of the cell models used (i.e., primary cortical neurons versus stem cell-derived i^3^Neurons) or the nature of the BioID2 and APEX2 enzymes. The APEX2 is a proximity labeling enzyme similar in principle to BioID2 as both proteins involve biotin labeling in ∼10 nm labeling radius. A major difference is that APEX2 is a peroxidase, which requires adding hydrogen peroxide and accordingly biotinylates proteins in an ∼1 min time frame limiting the identification of potential interacting proteins to a cross-sectional snapshot, which can be useful to study dynamics of tau interactions. In contrast, BioID2 labels proximal proteins and labeled proteins can accumulate over an extended amount of time (e.g., 4 days used here).

Two recent studies utilized the BioID2 approach to identify potential tau interactors.^28,29^ Prikas and colleagues expressed BioID2-Tau (2N4R) in primary neurons, mouse cortex, and hippocampus and in the hippocampus of TKO mice. We found significant differences between the proteins identified in our study and those identified in each model utilized in the other study. It is difficult to pinpoint the underlying reasons for the discordant findings, but it is likely that technical differences between the two studies may play a role. For example, the choice of expression vectors (lentiviral versus adeno-associated viruses) and competition with endogenous tau in wild-type primary neuron experiments (in the study by Prikas et al.) could have influenced results. Differences in the placement of BioID2 on tau could also alter the outcomes. We tagged BioID2 individually to the N-terminus and the C-terminus of tau, while Prikas et al. tagged BioID2 only to the N-terminus of tau. The labeling radius of the BioID2 protein is estimated at ∼10 nm,^31^ thus, BioID2 positioning could impact labeling of specific proteins. Moreover, we compared our list of identified proteins to those found by Griffin and colleagues using the BioID2 approach and found that only 16 proteins overlapped with our study.^29^ This could be explained in terms of the tau isoform used (2N4R versus 0N4R), the experimental differences associated with K18 fibril treatment of stem cell-derived NGN2 neurons, and again placement of the BioID2 enzyme.^29^ Griffin et al. tagged BioID2 to the C-terminus of 0N4R tau isoform.^29^ Additionally, differences in the criteria used to define tau protein interactors might influence the variability between the different interactome studies. Taken together, using traditional and *in situ* labeling approaches coupled with mass spectrometry produced a significant body of work highlighting potential functional interactions between tau and many other proteins, which require follow-up studies to better understand their potential roles in biology and pathobiology.

Enzyme-mediated proximity labeling approaches (e.g., BioID2) provide a complementary framework for identifying protein–protein interactions and overcome several of the limitations associated with the antibody-based approaches. However, some limitations remain that are worth considering. Creation of nonphysiologically fusion proteins as well as biotinylation of the fused target protein (i.e., tau) by the enzyme (i.e., BioID2 or APEX2) could potentially interfere with the protein interactions by altering the folding or the functionality of tau. The BioID2-tau proteins were expressed in TKO neurons to help mitigate the overexpression effects. However, lentiviral transduction may lead to tau overexpression in a tau null background, which may affect interactions or induce tau aggregation. While we did not observe evidence of tau aggregation in our cultures (i.e., high molecular weight tau in Western blots or visible aggregates in images), this remains a caveat that requires further investigation. Recently, we did not find evidence of pathological tau species in MAPT-KI mice out to 24 months of age.^32^ Thus, the validation of BioID2-Tau-identified interactions using PLA from MAPT-KI primary neurons and co-IPs from MAPT-KI brain tissue suggests overexpression and pathological tau species were unlikely to affect the results. Proximity labeling approaches are dependent on the labeling radius of the enzyme (∼10 nm) as well as the surface-exposed lysine residues available for labeling by the BioID2 enzyme. It is noteworthy that this allows for the identification of weak/transient protein interactions; however, this could lead to missing a potential interactor protein (>10 nm) or mislabeling proteins that are within the vicinity of the labeling radius but are not directly interacting with the target protein. Finally, these approaches do not effectively confirm or eliminate the possibility that positively identified proteins are part of a multimeric complex with intermediate proteins bridging tau and the labeled protein.

## Conclusion

The results presented in this study provide evidence of the myriad functions of the human tau protein. The diverse interactome of tau across several cellular compartments and pathways is consistent with its widespread distribution in neurons and the high flexibility of disordered proteins, which allows for interactions with multiple binding partners often in a context-dependent fashion. This work also demonstrates the utility of the BioID2 approach coupled with complementary validation approaches in providing an effective route to identifying potential tau protein interactors. Future studies could pursue the molecular mechanisms and biological functions related to the potential tau protein interactors identified here and in prior studies. This could reveal novel roles for tau in various physiological functions and may inform potential pathways that are dysregulated by tau abnormalities in tauopathies.

## Supplementary Material

















## Data Availability

All the data are provided in this article, the Supporting Information, and/or on Dryad. Mass spectrometry output files (.RAW, mzML, and .mzID) generated in this study are deposited to the open access DRYAD repository, 10.5061/dryad.280gb5mxj. Any additional information is available upon reasonable request to Dr. Nicholas Kanaan (corresponding author) at nkanaan@msu.edu.

## Notes

Supplementary methods; Figure S1. Nontransduced tau knockout primary cortical neurons immunofluorescence; Figure S2. Full representative Western blot images for blots in Figures 1, 4, 5, and 6; Figure S3. Qualitative analysis of candidate tau interactors identified in three-out-of-three replicates; Figure S4. Comparing candidate tau interactors identified in this study to a comprehensive tau interactome review and three enzyme-mediated proximity-labeling approaches previously reported in the literature; Figure S5. Primary antibody deletes for PLA in human MAPT KI neurons (PDF)
Table S1. Tau interacting proteins identified in this study using qualitative and LFQ analyses (related to Figure 2) (XLSX)
Table S2. Peptide lists for proteins identified with qualitative and LFQ analyses (XLSX)
Table S3. Tau interacting proteins identified in three-out-of-three replicates (related to Figure S3) (XLSX)
Table S4. GO Cellular component, GO molecular function, GO Biological process, and KEGG pathways analyses of the identified candidate tau interactors (related to Figure 3) (XLSX)
Table S5. Candidate tau interactors unique to this study compared to a comprehensive tau interactome review and three enzyme-mediated proximity-labeling approaches previously reported in literature (related to Figure S4) (XLSX)
Table S6. Criteria used to identify tau protein interactors in published tau interactome studies (XLSX)
Table S7. GO analyses (related to Figure 4B, Figure 5B, and Figure 6B) (XLSX)

## #

Structural Biology Department, Van Andel Institute, Grand Rapids, MI, United States

## Abbreviations

AD: Alzheimer’s disease
BioID2: biotin identification
co-IP: coimmunoprecipitation
MAPT: microtubule-associated protein tau
APEX2: ascorbate peroxidase
LFQ: label-free quantification
PLA: proximity ligation assay
MAPT KI: Human MAPT (tau) knock-in
TKO: tau knockout
DMEM: complete Dulbecco’s modified eagle medium
FBS: fetal bovine serum
E18: embryonic day 18
CMF: calcium- and magnesium-free solution
SDS: sodium dodecyl sulfate
TBS: Tris-buffered saline
TBS-T: Tris-buffered saline with Tween-20
PFA: paraformaldehyde
BSA: bovine serum albumin
DAPI: 4′,6-Diamindino-2-Phenylindole
ACN: acetonitrile
FDR: false discovery rate
STRING: search tool for the retrieval of interacting genes/proteins
MCL: Markov clustering
GO: gene ontology
PP1: protein phosphatase 1.
