# Supplementary material for: BioID2-Based Tau Interactome Reveals Novel and Known Protein Interactions Associated with Multiple Cellular Pathways
Source: J Proteome Res. 2025 Sep 5;24(10):5099–115. doi: 10.1021/acs.jproteome.5c00473 (PMC12495503; doi:10.1021/acs.jproteome.5c00473)
Supplement: Supplementary file 1 [file pr5c00473_si_001.pdf]

SUPPLEMENTAL DATA

BioID2-based tau interactome reveals novel and known protein interactions associated with multiple cellular pathways

Ahmed Atwa[1,2], Mohammed M. Alhadidy[1,2], Jared Lamp[1,3], Benjamin Combs[1], and Nicholas M. Kanaan[1,2,*]

Table of Contents:

1. Supplementary methods pages 2-4
2. Supplemental Figures pages 5-10
3. Supplemental Table S1. Tau interacting proteins identified in this study using qualitative and LFQ analyses (XLSX)
4. Supplemental Table S2 Peptide lists for proteins identified with qualitative and LFQ analyses (XLSX)
5. Supplemental Table S3 Tau interacting proteins identified in three out of three replicates (XLSX)
6. Supplemental Table S4 GO Cellular component, GO molecular function, GO Biological process, and KEGG pathways analyses of the identified candidate tau interactors (XLSX)
7. Supplemental Table S5 Candidate tau interactors unique to this study compared to a comprehensive tau interactome review and three enzyme-mediated proximity-labeling approaches previously reported in literature (XLSX)
8. Supplemental Table S6 Criteria used to identify tau protein interactors in published tau interactome studies (XLSX)
9. Supplemental Table S7 GO analyses (XLSX)

Supplementary Methods

Cloning of the BioID2 Constructs

Site-directed mutagenesis

Single-site and multi-site mutagenesis reactions were performed using QuikChange Lightning site-directed mutagenesis Kit (Agilent Technologies, #210518 and #210516) per the manufacturer's instructions. The BioID2-HA plasmid was mutated to include a KpnI restriction site upstream of the BioID2 sequence and to include an EcoRV site and a Kozak sequence upstream of the KpnI site. Then a NotI restriction site was inserted downstream of the BioID2 sequence. The following primers were used:

- KpnI site insertion:
  5'- cggattcgaattcggatccGGTACCttttcggaattcggatccg-3'
- EcoRV site and Kozak sequence insertion:
  5'-cggtcgtacgtctccgGATATCAAGCCACCATGggtaccttttcg-3'
- NotI site insertion: 5'-gatgtaccggattacgcatagGCGGCCGCcgctgatcagcctc-3'

The Myc-BioID2 plasmid was mutated to insert an EcoRV restriction site upstream of the BioID2 sequence and an NdeI restriction site downstream of the BioID2 sequence, and to delete the endogenous NdeI restriction site upstream of the BioID2 sequence. The following primers were used:

- EcoRV site insertion: 5'- cactatagggagacccaagcGATATCgccaccatggaac-3'
- NdeI site insertion: 5'- gtggatcggcgcgccgtCATATGaacctcgagc-3'
- NdeI site deletion: 5'- gcagtacatcaagtgtatcCGCGgccaagtacgccccctattgacg-3'

Restriction Digestion

Digestion reactions were prepared by adding 500 ng plasmid DNA samples, 1 µl of each restriction enzyme, 2 µl fast digest green buffer and nuclease-free H_2O to a total volume of 20 µl. Digestion reaction was incubated for 1 hour at 37°C. Restriction enzymes used for the plasmids created were fast digest EcoRV (ThermoScientific, #FD0303), KpnI

(ThermoScientific, #FD0524), NdeI (ThermoScientific, #FD0584), NotI (ThermoScientific, #FD0596), and XhoI (ThermoScientific, #FD0694).

Bacterial Transformation

For each transformation reaction, XL10-Gold ultracompetent cells or *Stbl3 competent E.coli* (ThermoScientific, #C7373-03) were thawed on ice and DNA was added to the cells and left for 10 minutes. Cells were heat-shocked for 30 seconds at 42 °C and left on ice for 2 minutes. Pre-heated LB broth (360 µl) was added to the bacterial cells and incubated for 1 hour at 37 °C with shaking at 300 rpm. The entire culture was poured on LB/agar plate, streaked to cover the plate, and incubated at 37 °C for 12-16 hours.

Preparation of LB/agar plates and LB broth medium

The LB/agar was prepared by dissolving 10 grams of LB broth base (Sigma-Aldrich, #L3522), 7.5 grams of Agar (Sigma-Aldrich, #A1296) in 500 ml double-distilled water (ddH_2O) and autoclave using a liquid cycle. LB/agar was cooled at room temperature, 50 mg ampicillin (Sigma-Aldrich, #A9518) were added while still warm, and approximately 20 ml of LB/agar were poured into petri dishes and left at room temperature to solidify. LB/agar plates were used directly or stored at 4°C. LB broth base was prepared by dissolving 10 grams of LB in 500 ml ddH_2O.

Plasmid DNA Miniprep

Single colonies (5-10 colonies/construct) from the streaked LB/agar plates were picked using a micropipette tip and inoculated into a culture of 5 ml LB containing ampicillin (100 µg/ml) in a sterile glass test tube and incubated for 12-16 hours at 37 °C with shaking at 225 rpm. Bacterial cells were centrifuged at 8000 x g for 3 minutes at room temperature and supernatant was discarded. Plasmid DNA extraction was performed using QIAprep spin miniprep kit (Qiagen, #27104) following the manufacturer's instructions. Plasmid DNA concentration was measured using NanoDrop spectrophotometer

(ThermoScientific, #ND-2000). Validation of plasmid DNA was done by both restriction digestion and Sanger sequencing (Genewiz).

Plasmid DNA Maxiprep

A single colony was picked from LB/agar plate, inoculated into a 5 ml LB sterile glass test tube containing ampicillin (100 µg/ml), and incubated overnight at 37 °C with shaking at 225 rpm. The 5ml bacterial culture was inoculated into 250 ml LB containing ampicillin (100 µg/ml) in a flask. The bacterial culture was left to grow overnight at 37 °C with shaking at 225 rpm. The bacterial cells were harvested by centrifugation at 6,000 x g for 15 minutes at 4 °C, and the plasmid DNA maxiprep was performed using the Qiagen plasmid maxi kit (Qiagen, #12162) following the manufacture's protocol.

DNA Gel Electrophoresis

One liter of 50X TAE buffer stock solution was prepared by adding 242.28 g Tris-base (Sigma-Aldrich, #10708976001), 57.2 ml glacial acetic acid, 100 ml of 500 mM EDTA buffer (pH 8.0) and adjusting the volume to one liter by adding ddH_2O. One liter 0.5X TAE buffer working solution was prepared by adding 10 ml 50X TAE buffer to 990 ml ddH_2O. Agarose gel (1%) was prepared by dissolving 2 grams of agarose (Bio-Rad, #1613101) in 200 ml 0.5X TAE buffer and microwaving for 2-4 minutes until fully dissolved, and adding 20 µl ethidium bromide (Bio-Rad, #1610433). The agarose gel was poured into a gel electrophoresis system (ThermoScientifc, #09-528-110B), and left to polymerize for 30-40 minutes at room temperature. 20 µl of 1kilobase DNA ladder (NEB, #N3232S) was added to the first well then 20 µl digestion reaction was added. The gel was run at 150 constant volts for 45-60 minutes and the DNA bands were visualized under a UV transilluminator (Accuris, #E3100).

Gel purification and DNA ligation

The DNA gels were placed on a UV transilluminator, and the desired DNA band was cut out using a razor blade and placed it into a microcentrifuge tube. DNA gel extraction was performed using QIAquick Gel Extraction Kit (Qiagen, #28706) following manufacturer's instructions. Then, the ligation reaction was prepared by adding 2 µl of open plasmid backbone, 8 µl of the desired insert (with compatible overhangs), 1 µl of 10X T4 buffer, 1 µl of T4 DNA ligase enzyme (ThermoScientific, #EL0011), adjusting the final volume to 20 µl with ddH_2O, and incubating overnight at 16 °C. The ligation reaction was used to transform a bacterial strain, purified using QIAprep spin miniprep kit (Qiagen, #27104), and validated by restriction digestion and Sanger sequencing (Genewiz).

Lentiviral functional titer determination using Immunocytochemistry (ICC)

HEK293T cells were plated in a 24-well plate at a density of 75,000 cells/well and incubated overnight. Cells were transduced by lentiviruses expressing either the fusion proteins Tau-BioID2, BioID2-Tau or the respective controls BioID2-HA, and Myc-BioID2. Cells were transduced by lentiviruses at 10-fold dilutions from 1:10^2 to 1:10^9 and maintained for 72 hours in a humidified incubator. Cells were fixed in 4% paraformaldehyde (Electron Microscopy Sciences, #15714) prepared in 1x cytoskeleton buffer (10 mM MES, 138 mM KCl, 3 mM $MgCl_2$, 4 mM EGTA pH 6.1, all from Sigma-Aldrich). Cells were rinsed four times by 1x TBS and incubated for 1 hour at room temperature in blocking buffer containing 5% goat serum (GS, VWR, #10152-212),1% BSA, 0.2% Triton-X100 (Bio-Rad, #161-0407) prepared in 1x TBS. Cells were incubated in primary antibodies diluted in 2% GS-1x TBS and maintained overnight at 4 °C. Cells were rinsed four times by 1x TBS and were incubated for one hour in biotinylated secondary antibodies diluted 1:500 in 2% GS. Avidin-biotinylated horseradish peroxidase (HRP) complex (ABC, ThermoScientific, #32020) was prepared by adding 1 drop of each reagent A and B to 10 ml 1x TBS and incubating for 30 minutes at 4 °C. Cells were rinsed four times in 1x TBS and were incubated in ABC solution or 1 hour at room temperature. The peroxidase substrate was prepared by adding 2.5 mg DAB (Sigma-Aldrich, #281751) to 5 ml 1x TBS and then adding 0.5 µl 30% H_2O_2. Cells were rinsed four times in 1x TBS and the peroxidase substrate was added for 5 minutes at room temperature. The assay

development was stopped by removing the substrate and rinsing four times in 1x TBS. Cells were visualized under inverted light microscope Nikon eclipse TE2000-U. The lentiviral functional titer was calculated by multiplying the number of transduced cells at the highest viral dilution by the dilution factor. Primary antibodies used were HA-tag (1:4000, Cell Signaling Technology, #3724, RRID: AB_1549585) and Myc-tag (1:4000, Cell Signaling Technology, #2276, RRID: AB_331783). Secondary antibodies used were Biotinylated goat anti-mouse IgG (Jackson ImmunoResearch Labs, #115-065-166, RRID: AB_2338569) and biotinylated goat anti-rabbit IgG (Vector Laboratories, #BA-1000, RRID: AB_2313606.

Supplemental Figures

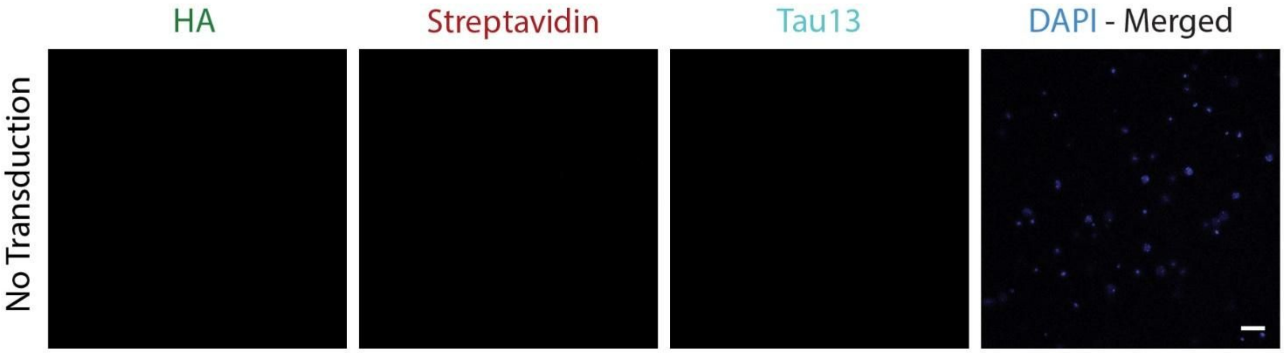

FIG.S1. **Non-transduced tau knockout primary cortical neurons for biotinylation and human tau staining controls.** showing no expression of the BioID2 proteins (HA, green channel), no expression of human tau (Tau13, cyan channel), and no signal in red streptavidin channel confirming that the biotinylation signal observed in Fig.1*C* and *D* is from the proximity-dependent biotinylation by the BioID2 proteins rather than being endogenously biotinylated. Scale bar 25 μm.

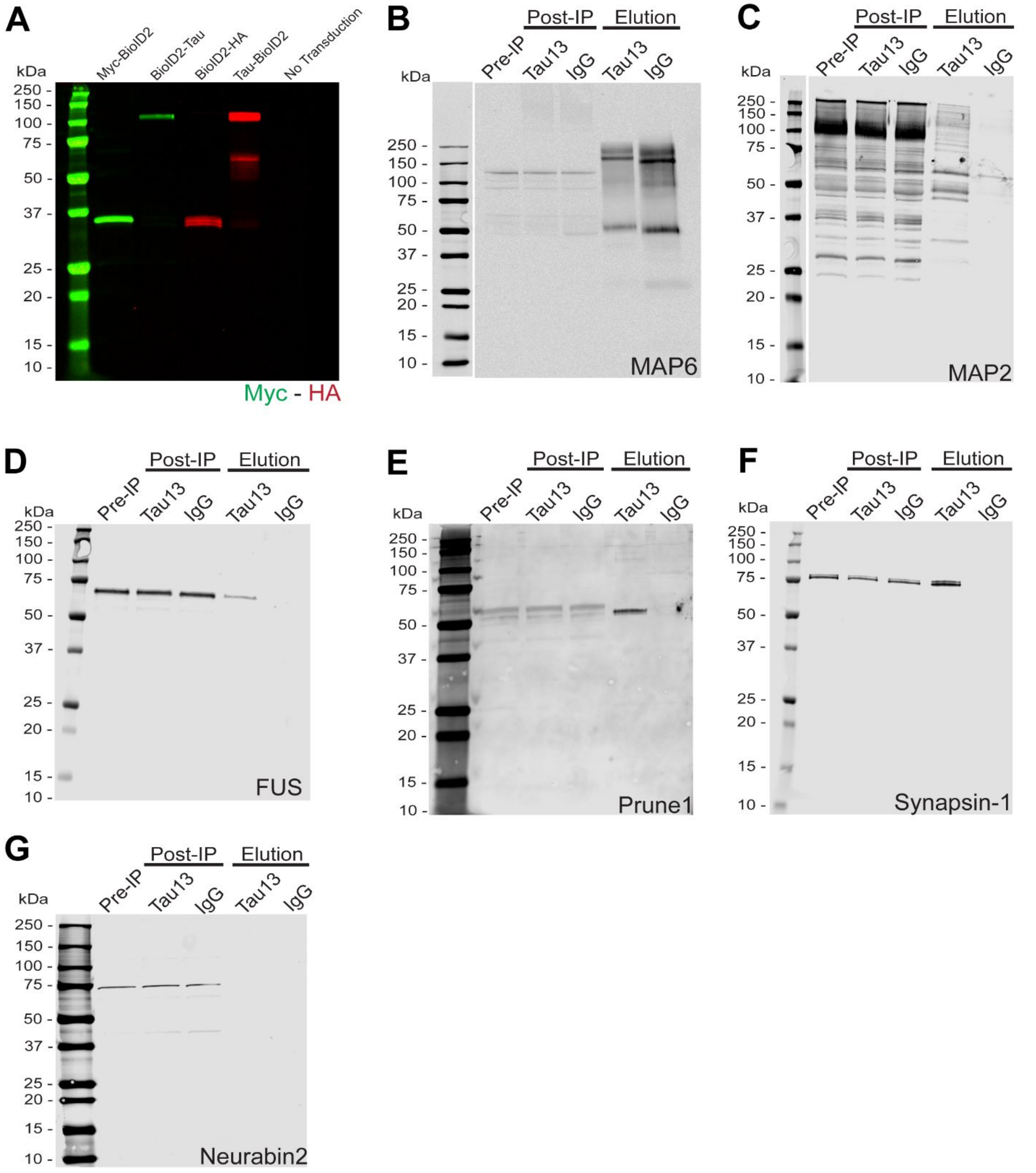

FIG.S2. **Full membranes of representative western blot images in Figures 1, 4, 5, and 6.** *A,* Full representative blot of lentiviral expression in TKO primary cortical neurons from Fig. 1B. *B,* Full representative blot of MAP6 and tau co-IP from Fig. 4E (ladder was captured using a different channel on the LiCor imaging system). *C,* Full representative blot of MAP2 and tau co-IP from Fig. 4H (ladder was captured using a different channel

on the LiCor imaging system). *D,* Full representative blot of FUS and tau co-IP from Fig. 5E. *E,* Full representative blot of Prune1 and tau co-IP from Fig. 5H. *F,* Full representative blot of Synapsin-1 and tau co-IP from Fig. 6E. *G,* Full representative blot of Neurabin2 and tau co-IP from Fig. 6H. TKO, Tau knockout; Pre-IP indicates the lysate; post-IP indicates the supernatant; co-IP, co-immunoprecipitation

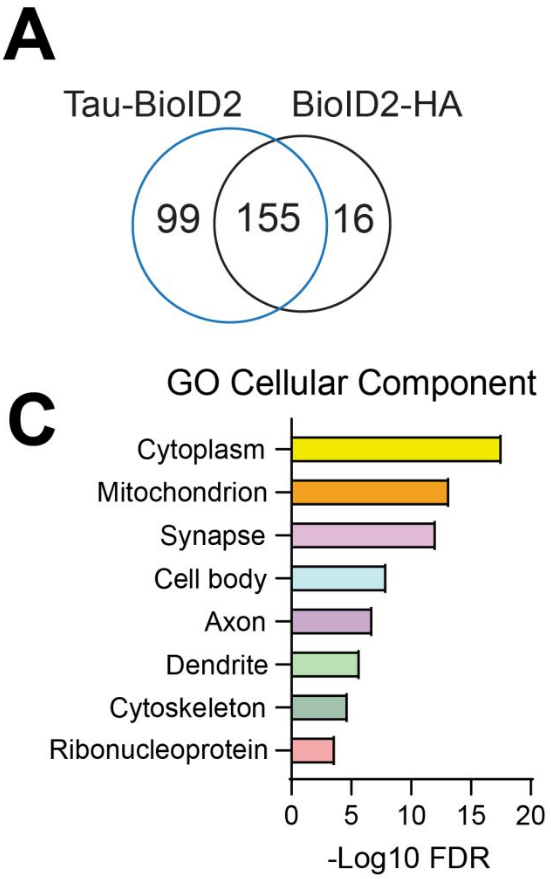

FIG.S3. **Qualitative analysis of candidate tau interactors identified in three-out-of-three replicates.** A, Analysis using more stringent criteria for the mass spectrometry data in three-out-of-three experimental replicates identified 99 proteins as potential interactors with Tau-BioID2 compared to the BioID2-HA control. B, This analysis identified 41 proteins as potential interactors with BioID2-Tau compared to the Myc-BioID2 control. C, GO cellular component analysis of the identified proteins includes annotations with a false discovery rate ≤0.05. Selected annotations were graphed with -log10(FDR). The full list of annotations is in Supplemental Table S3. D, comparing the two-out-of-three versus

three-out-of-three analyses resulted in 78 proteins overlapping between both analyses, 144 proteins identified in the two-out-of-three analysis, and 43 proteins identified in the three-out-of-three analysis. The full list of proteins is in Supplemental Table S3.

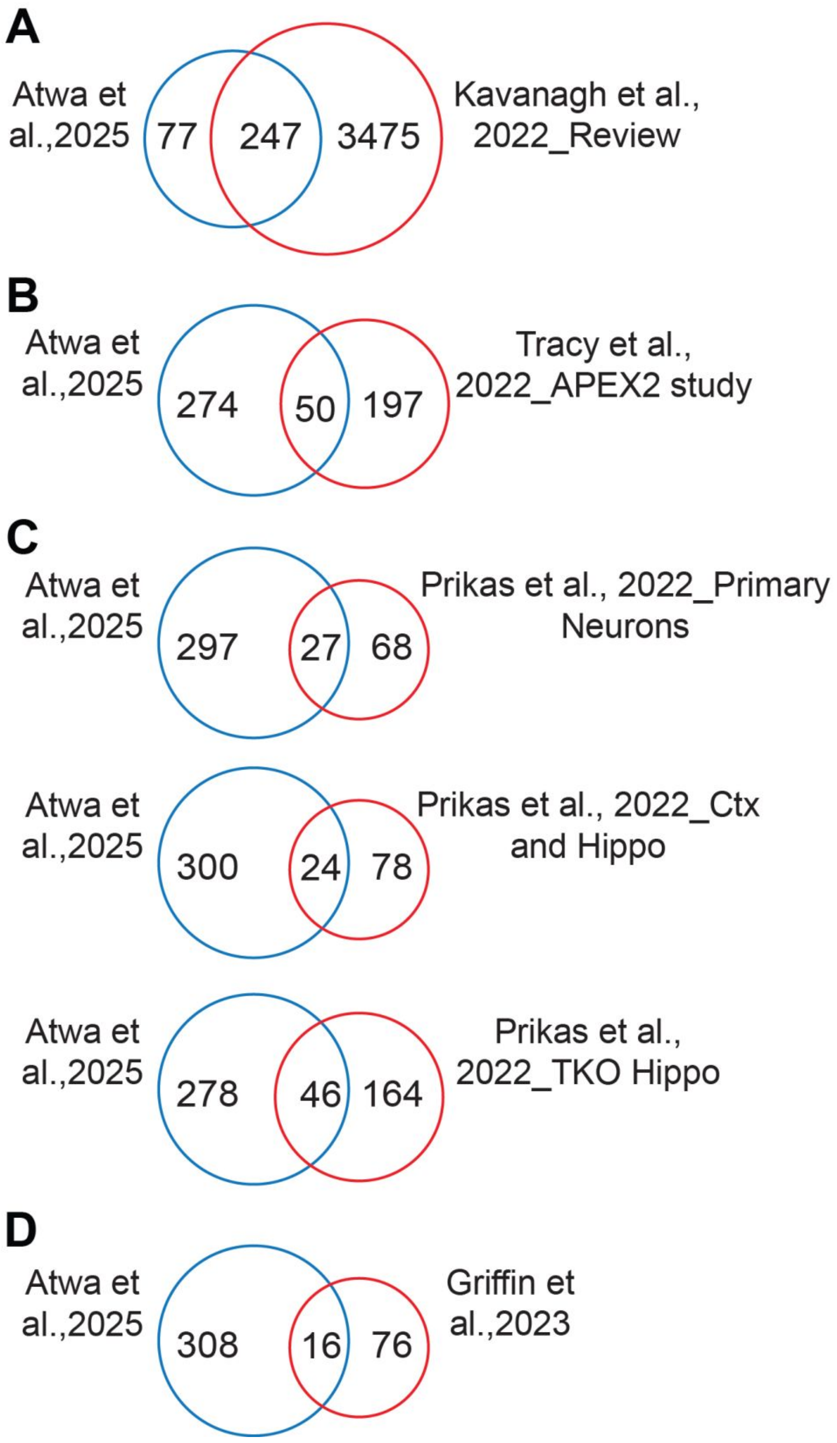

FIG.S4. **Comparing the proteins identified in this study to a comprehensive tau interactome review and three enzyme-mediated proximity-labeling approaches**

previously reported in literature. *A,* Venn diagram showing the number of proteins overlapped (247 proteins) between this study and the comprehensive tau interactome review that included 12 tau interactome studies previously reported, 77 proteins were uniquely identified in this study. *B,* Venn diagram showing the number of proteins overlapping between this study and the APEX2-based approach. *C,* three Venn diagrams showing the overlapping number of proteins between this study and the three different models expressed BioID2-Tau (2N4R) in primary neurons, *in vivo* in cortex and hippocampus, and *in vivo* in hippocampus of 6-month-old TKO mice. *D,* Venn diagram showing the number of proteins identified in this work and (0N4R) Tau-BioID2 expression in stem cell-derived NGN2 neurons. Ctx; cortex, Hippo; hippocampus, TKO; mouse tau knockout.

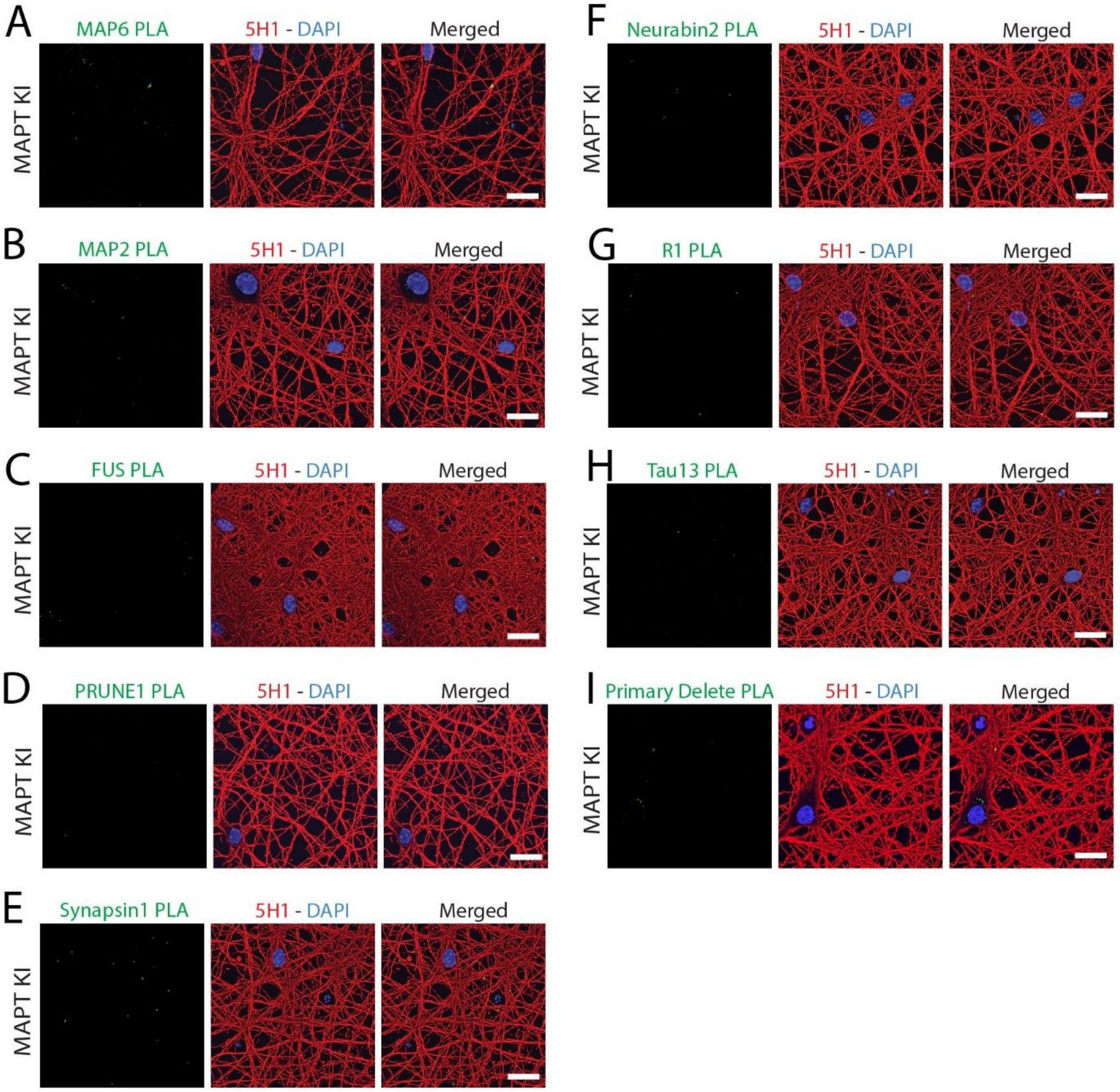

FIG.S5. **Primary antibodies delete PLA in human MAPT KI neurons.** Negative controls showing little to no PLA puncta in PLA performed in MAPT KI primary cortical neurons using only one antibody. *A,* MAP6 only PLA, *B,* MAP2 only PLA, *C,* FUS only PLA, *D,* prune1 only PLA, *E,* synapsin-1 only PLA, *F,* neurabin-2 only PLA, *G,* R1 only PLA, *H,* Tau13 only PLA, *I,* no primary antibodies PLA. Tubulin immunofluorescence shown in red channel (5H1) and nuclei were labelled by DAPI (blue). Scale bar 20 µm. PLA; proximity ligation assay, MAPT KI; human MAPT knock-in.
